# Return-to-Play Criteria Following Lower Limb Muscle Injuries in Soccer: A Systematic Review with Evidence Synthesis

**DOI:** 10.1007/s40279-026-02404-9

**Published:** 2026-03-18

**Authors:** Javier Pecci, Nicol van Dyk, Gregory D. Myer, Borja Sañudo

**Affiliations:** 1 Department of Physical Education and Sport, University of Seville, Seville, Spain; 2 Section Sports Medicine, Faculty of Health Sciences, University of Pretoria, Pretoria, South Africa; 3 School of Public Health, Physiotherapy and Sport Sciences, University College Dublin, Dublin, Ireland; 4 Sports Performance And Research Center (SPARC), Emory University School of Medicine, Flowery Branch, GA USA; 5 Department of Orthopaedics, Emory University School of Medicine, Atlanta, GA USA; 6 Wallace H. Coulter Department of Biomedical Engineering, Georgia Institute of Technology and Emory University, Atlanta, GA USA

## Abstract

**Objective:**

The objective was to systematically identify and categorize return-to-play (RTP) domains and criteria used following muscle injuries in male soccer players, and to describe the certainty of the evidence according to the number, design and methodological quality of the studies implementing RTP criteria within each domain.

**Methods:**

In total, six databases were searched up to 10 March 2024. Studies reporting RTP criteria for hamstring, adductor, quadriceps, and calf injuries, as well as general criteria for all muscle injuries, were included. The certainty of evidence for RTP criteria was assessed on the basis of the studies citing each criterion.

**Results:**

Out of 58,057 records, 135 studies met the inclusion criteria. Strength and pain criteria are the most cited tests for determining RTP clearance following hamstring injuries, particularly criteria related to between-limb knee flexors/extensors strength symmetry and no pain during soccer-specific actions. Range of motion criteria (active knee extension, passive and active straight leg raise, and Askling-H tests) and subjective readiness demonstrated the highest certainty of evidence in RTP decision making after hamstring injuries. RTP criteria following adductor injuries showed a moderate-to-very-low certainty of evidence across domains. Criteria for quadriceps and calf injuries ranged from low to very low evidence, while general lower limb muscle injury criteria had only very low evidence. Following adductor injuries, the highest evidence (moderate) was shown by pain assessments and completing at least one full team training session.

**Conclusions:**

Symmetry between limbs in knee flexor and extensor strength combined with no pain during soccer-specific actions were the most frequently implemented RTP criteria for hamstring injuries. In addition, range of motion evaluation (i.e., active knee extension, passive and active straight leg raise, and Askling-*H* tests) and subjective readiness assessments have been more consistently employed in higher-quality intervention studies than other domains following hamstring injuries in soccer players. Pain assessments, completing at least one full team training session and restoring strength levels are the most cited criteria for RTP following adductor injuries. The evidence base describing RTP criteria for quadriceps and calf injuries is limited and is not implemented in randomized controlled trials with high methodological quality, highlighting the need for further robust research in these domains.

**Trial Registration Number:**

PROSPERO CRD42022363836.

**Supplementary Information:**

The online version contains supplementary material available at 10.1007/s40279-026-02404-9.

## Key Points

**Table:** 

Strength and pain were the most frequently cited return-to-play (RTP) criteria following hamstring injuries, particularly symmetry in knee flexor/extensor strength and absence of pain during soccer-specific drills. Range of motion and subjective readiness criteria were also commonly used and have been implemented in a relatively larger number of higher-quality randomized controlled trial studies.
Pain assessments and completion of at least one full team training session were the RTP criteria most implemented and described in higher-quality intervention studies following adductor injuries.
Strength assessments for adductor injuries and muscle imaging for quadriceps and calf injuries were frequently reported in a notable number of studies.
Following quadriceps and calf injuries, pain assessment, muscle imaging without swelling, and completion of a full training session were the RTP criteria most often implemented, but the overall evidence base remains limited and is not implemented in high-quality randomized controlled trials.

## Introduction

Although the total number of injuries have decreased in recent years for soccer players, muscle injury incidence has not changed [1]. Muscle–tendon injuries constitute a severe problem, presenting high costs and burden for professional soccer teams [2]. In addition to the high prevalence (i.e., about one-third of all injuries in soccer) [3–5], the elevated risk of re-injury associated with these injuries compared with other soccer-related injuries [4, 5] makes rehabilitation particularly challenging. Further, re-injury burden is usually associated with a longer time to return-to-play (RTP) compared with index injuries [4, 5], which complicates the successful return to competition for players. Injury epidemiology studies report that 90% of all muscle injuries involve the four main lower limb muscle groups: hamstring, quadriceps, adductors, and calf [5, 6]. Among these, hamstring injuries are the most frequent (37%), followed by adductors (23%), quadriceps (19%), and calf (13%) [5].

Muscle injuries lead to deficits in the minutes played [7, 8], maximum velocity reached during matches [7, 9], high-speed running performance [10, 11], and high-intensity accelerations and decelerations in competition [8]. Consequently, while we should focus on reducing re-injury risk, we should also work on recovering soccer player performance. Numerous tests can assess player performance, providing information about risk of injury recurrence throughout rehabilitation [12–14]. However, there is a lack of consensus on the evidence for different tests to be included that ensure a safe and time-efficient RTP.

Several studies have tried to improve traditional rehabilitation protocols by designing criteria-based progressions in each phase of the rehabilitation process [15–18]. A critical moment during the rehabilitation process is deciding whether the player is ready or not for RTP [19]. Diverse RTP criteria have been proposed to determine clearance after muscle injury [20–22]. However, despite growing evidence on RTP, current clinicians’ rehabilitation practices do not perform the objective criteria recommended from previous research to guide RTP decision-making [23, 24]. The increasing body of literature reporting RTP criteria needs a quantitative and qualitative (e.g., certainty of evidence for studies citing each criterion) approach for guiding clinicians in the decision-making for clearance. In addition, to the author’s knowledge, no studies to date have collected all available evidence on RTP criteria following muscle injuries, especially for adductors, quadriceps and calf.

Therefore, the objective of this systematic review was to systematically evaluate the literature on RTP criteria following muscle injuries in soccer players and summarize the evidence in a quantitative and qualitative approach.

## Methods

This systematic review was pre-registered (PROSPERO CRD42022363836) and followed Preferred Reporting Items for Systematic Review and Meta-Analysis (PRISMA) [25] and PRISMA in Exercise, Rehabilitation, Sport Medicine and Sports science (PERSiST) guidance [26].

### Search Strategy

In total, six databases (PubMed, Web of Science, SPORTDiscus, CINAHL, Embase, and PsycInfo) were explored from database start to 10 March 2024. Each database was subjected to specific search strategies outlined in Online Supplementary File 1, encompassing key terms, search date, and search equation. Following the identification of duplicates, two authors (J.P. and B.S.) independently screened titles and abstracts in a first screening round and full text in a second screening round. Disagreements at the end of the screening process were resolved by discussion with the remaining two authors (N.V.D. and G.D.M.). Reference lists from the included studies were also screened to detect potential eligible studies.

### Eligibility Criteria

*Study design*: Studies included in this review comprised those that reported RTP criteria implemented in rehabilitation programs following lower limb muscle injuries in soccer players. Additionally, to gather all available evidence on RTP criteria, studies identifying potential criteria through cross-sectional retrospective analyses (e.g., comparing deficits in previously injured versus non-injured players), and expert consensus, surveys or interviews were also considered for extracting all information about RTP criteria. The inclusion of cross-sectional studies and expert consensus statements represented a deviation from the original PROSPERO protocol, which had initially specified the inclusion of interventional studies only. This additive information is reported in the supplementary materials (Supplementary File 3), keeping the review primarily focused on interventional studies [27]. Therefore, no restrictions were imposed on study design, except for excluding systematic reviews and meta-analyses; therefore, only original studies were included. No restrictions were applied for language, using translation tools when necessary and studies in abstract form were excluded.

*Participants:* Eligibility criteria were tailored to the study design. For interventional studies (and other designs reporting data from individual athletes undergoing rehabilitation), studies had to include male or female competitive soccer players aged ≥ 16 years, regardless of competitive level, and report RTP criteria. Regarding the injury type and location for interventional studies, acute myofascial and musculotendinous injuries [28] to the hamstrings were included, while tendinous injuries were excluded. Therefore, studies that reported damage solely on tendon tissue such as tendinopathies, type C injuries in the British Athletics Muscle Injury Classification (BAMIC) classification system [28], complete tendon ruptures reported as type 4 injuries in the Munich Consensus classification system [29] or avulsions were not included, since the implications of the injury might be substantially different [30, 31]. Specifically for adductor injuries, the classification of groin injuries was primarily based on the Doha agreement on terminology and definitions of groin pain [32]. Accordingly, only acute adductor-related muscle injuries were included in this review. Chronic conditions such as chronic adductor-related groin pain, characterized by persistent symptoms over time, were excluded. All studies were considered eligible if they included soccer players, regardless of the percentage that soccer players represented within the total sample. To enhance transparency, Supplementary File 2 specifies both the total number of participants in each study and the number of soccer players represented. However, a sensitivity analysis excluding studies with < 50% soccer players in their sample was performed to verify whether the more comprehensive inclusion affected the certainty of evidence ratings or the conclusions of the present review. For expert consensus statements, cross-sectional studies, surveys, or interviews that did not recruit individual participants undergoing rehabilitation, eligibility was based on whether the proposed RTP criteria were explicitly oriented towards soccer players with the same types of injuries and demographic characteristics as those included in the interventional studies. We also excluded studies with surgically treated injuries.

*Interventions:* For interventional studies, studies implementing rehabilitation programs following muscle injuries were included. For the remaining studies, no restrictions were applied for intervention criteria.

*Outcomes*: The outcomes included RTP criteria following lower limb muscle injuries.

### Data Extraction and Synthesis

Two authors (J.P. and B.S.) independently extracted data from the included studies, reporting title, authors, publication year, sample size, gender distribution (male and female soccer players), age, height, body mass, soccer exposure (training and matches), competitive level, study design, injury definition, dominant limb affected, injury location and severity, mechanism of injury, measured outcomes, details of rehabilitation programs including progression criteria, definition of RTP, time to RTP for both intervention and control groups, significant results with corresponding *p*-values, summary of findings, injury rate, number of re-injuries, re-injury rate, risk of re-injury (for intervention studies only), conclusions, and RTP criteria.

Two authors (J.P. and B.S.) classified RTP criteria, with any discrepancies resolved through discussion with the remaining two authors (N.V.D. and G.D.M). RTP criteria were categorized into groups, each comprising one to eight domains based on existing evidence.

### Certainty of Evidence for Studies Reporting RTP Criteria

This systematic review had two main objectives: (1) to map and categorize the RTP criteria and domains used following muscle injuries in male soccer players and (2) to describe, using the Grading of Recommendations Assessment, Development and Evaluation (GRADE) approach, the certainty of the evidence base for each RTP domain by summarizing the number, design and methodological quality of the studies that implemented at least one criterion within that domain. This evaluation was intended to characterize how well each domain has been investigated (i.e., the number, design, and methodological quality of the studies from which each criterion originates), rather than determine the effectiveness of specific RTP criteria for reducing reinjury risk or time to RTP.

Two authors (J.P. and B.S.) independently characterized the body of evidence available for each domain by summarizing the number and design of studies (e.g., randomized controlled trials, prospective cohort studies, retrospective analyses, case series, expert consensus) that implemented at least one RTP criterion within that domain. When applicable, we used the GRADE framework [33] to describe the overall certainty of the evidence base (i.e., our confidence in the consistency and completeness of the available studies describing each domain). Agreement was reached by author consensus. Given the nature of the assessed outcome (i.e., RTP criteria), which constitutes a part of the entire rehabilitation process and thus does not determine by itself the key outcomes in rehabilitation studies such as time to RTP or re-injuries, a modification of the GRADE classification was used [34]. This modified approach enables the application of GRADE classification to outcomes without meta-analyses and single effect estimate, allowing the certainty of evidence to be assessed in a more narrative manner [34] (see Supplementary File 4). The certainty of evidence was rated as high, moderate, low, or very low [33]. The baseline certainty of evidence was initially categorized as high if the suggested domain was reported in at least one randomized controlled trial (RCT). When no RCTs were available but the domain was reported in longitudinal cohort studies, the initial quality of evidence was classified as low [33, 35]. Subsequently, the certainty of evidence for each domain was downgraded by one or two levels for the following reasons on the basis of the GRADE approach and previously established criteria [33, 35, 36]. (1) Risk of bias: the certainty of evidence was downgraded by one level if high risk of bias was found in most included studies (> 50%) or by two levels if all studies included in a domain presented high risk of bias [33]. Risk of bias was assessed through the Risk of Bias 2 [37] for RCTs and Scottish Intercollegiate Guidelines Network Checklist 3 for longitudinal cohort studies [38] as previously reported in rehabilitation studies [35, 36]. Two authors (J.P. and B.S.) independently evaluated the risk of bias, with any discrepancies resolved through discussion with the remaining two authors (N.V.D. and G.D.M.) to achieve consensus when needed. (2) Indirectness: a low risk of indirectness due to our predefined eligibility criteria and the classification of the RTP in specific domains, which guaranteed specificity of populations, interventions, comparators, outcomes and similarity in the rated criterion, was automatically assigned. (3) Risk of publication bias: the certainty of evidence was downgraded by one level when publication bias was suspected. (4) Inconsistency: the certainty of evidence was downgraded by one level if the results presented heterogeneity in the assessment of the criterion when more than one study were included for a specific outcome (e.g., RTP criteria specific domain such as “no pain at palpation”). For this domain, high clinical heterogeneity in terms of key features of the populations, interventions, comparators, and co-interventions were considered for rating inconsistency [36]. (5) Imprecision: the certainty of evidence was downgraded by one level if < 800 participants were included for a specific domain [39]. For domains lacking RCTs or longitudinal cohort studies and reported solely in cross-sectional studies, surveys, interviews, case, and/or expert consensus studies, the certainty of evidence was classified as very low.

## Results

### Study Selection

**Fig. 1:**
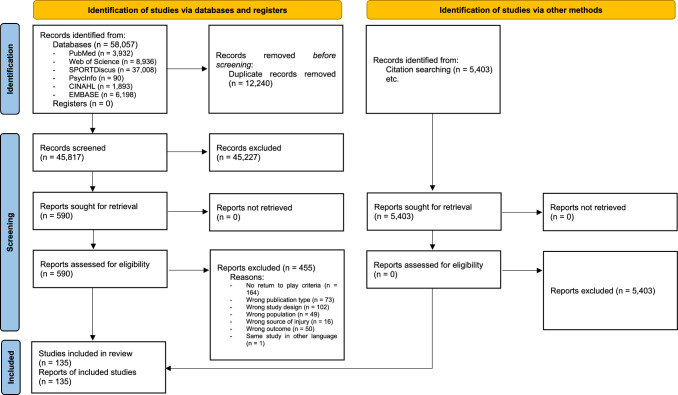
PRISMA flowchart of included studies

A total of 58,057 studies were identified through database searches. After removing duplicates (*n* = 12,240) and excluding irrelevant articles on the basis of title and abstract screening (*n* = 45,227), 590 studies remained for full-text assessment. Ultimately, 135 studies met the inclusion/exclusion criteria and proceeded with data extraction. Reference lists from included studies were screened to identify another potential 5403 studies for inclusion, although no additional eligible studies were found. The PRISMA flow diagram is presented in Fig. 1.

### Characteristics of Included Studies

Results of individual studies according to the data extraction process are presented in Supplementary File 2. Given the substantial number of studies reporting return-to-play (RTP) criteria for hamstring injuries (*n* = 86), the findings for this muscle group are presented separately from those of other muscle groups. Studies reporting RTP criteria on hamstring injuries were classified according to their experimental design: 13 RCTs [15, 40–51], 19 longitudinal cohort studies [10, 52–69], and 7 case studies [70–76]. In addition, evidence from 37 cross-sectional and/or retrospective studies [77–113], 4 expert consensus studies [13, 20–22], 2 surveys [23, 114] and 1 interview [115] is presented in Supplementary File 3. Two studies [116, 117] presented a cross-sectional retrospective analysis in addition to a longitudinal cohort prospective study, and one study [118] presented an expert consensus in addition to a longitudinal cohort design. Moreover, we identified studies with prospective follow-up [15, 41–45, 48, 49, 51, 53, 63, 66–69, 93, 97, 117]. Studies reported a total of 2844 (92%) male and 235 (8%) female soccer players. In addition, included studies reported 575 additional male athletes and 75 female athletes from studies reporting that soccer players were included from a broader cohort of athletes, but the specific number was not specified, and no information was received from the authors of these studies. Further, 57 studies included professional players, 25 studies included semi-professional players, and 21 studies included amateur/recreational players. All included studies were written in English.

RTP criteria for adductors injury were based on 22 studies [17, 20, 47, 61, 87, 92, 119–134], for quadriceps injury were based on 13 studies [11, 20, 45, 47, 61, 92, 135–141], and for calf injury were based on 9 studies [14, 18, 20, 47, 61, 142–145], whereas 12 studies reported RTP criteria for general lower limb muscle injury [9, 146–156]. According to experimental design, we identified 3 RCTs [45, 47, 125], 21 longitudinal cohort studies [11, 17, 18, 61, 92, 121, 122, 129–131, 134, 135, 140, 142, 146, 147, 149, 150, 152, 155, 156] and 7 case studies [119, 123, 132, 137–139, 143]. In addition, evidence from 13 cross-sectional and/or retrospective studies [9, 87, 120, 124, 126, 127, 136, 141, 144, 145, 148, 151, 153], 4 expert consensus studies [14, 20, 133, 154], and 1 survey [128] is presented in Supplementary File 3. We identified 14 studies with prospective follow-up [11, 18, 45, 47, 92, 121, 122, 129, 134, 142, 146, 150, 152, 155]. Studies specifically reported a total of 5817 male players and 1 female soccer player. Additionally, the studies included 127 more male athletes and 17 female athletes, including soccer players, though the exact number of soccer players was not specified. Despite requests, the authors did not provide this information. Regarding the competitive level, 33 studies [9, 11, 14, 18, 20, 61, 87, 92, 122–124, 128, 129, 131, 133–135, 137, 138, 140, 143, 144, 146–156] included RTP criteria for professional/elite players, 8 studies for semi-professional players [17, 87, 121, 126, 127, 132, 136, 141], and 6 studies [45, 47, 120, 139, 142, 145] for amateur players. In addition, one study [144] included professional and semi-professional players, while two studies [125, 130] included players from all three competitive levels.

### RTP Criteria

On the basis of available evidence, RTP criteria following hamstring injuries were classified as: (1) absence of pain, (2) external load progression, (3) jumping kinetics and kinematics, (4) strength, (5) range of motion, (6) biomechanics, (7) muscle activation, (8) muscle imaging, (9) rate of perceived exertion, (10) psychology, (11) healing, (12) soccer-specific ability, (13) muscle tissue properties, and (14) fitness (Table 1). RTP criteria were grouped into conceptual domains (e.g., strength, pain, range of motion, imaging, biomechanics, jumping kinetics and kinematics, muscle activation, and subjective readiness) on the basis of their primary construct and in line with two previous systematic reviews of RTP criteria on hamstring injuries [12, 158], one recent scoping review [194], as well as with expert consensus classifications [14, 20, 133]. In this context, the term “biomechanics” was used to encompass broader movement analyses, whereas “jumping kinetics and kinematics,” “strength,” and “muscle activation” were retained as separate domains to reflect commonly reported, discrete test categories (e.g., jump tests, strength testing, electromyographic assessments).

**Table 1:** Evidence-based return-to-play criteria following hamstring injuries used in included studies and certainty of evidence for the studies reporting each criterion

Return-to-play criteria	Number of studies reporting this criterion (*n* (%))	Certainty of evidence	Reinjury rate (range)	Mean time to return to play
Pain criteria	43 (50%)			
No pain at palpation [13, 15, 20, 40–43, 52, 66, 77, 113, 114]	12 (13.95%)	Low	0–23.07%	15–28.8 days
No pain during soccer-specific actions [13, 15, 20–23, 42–49, 51–56, 59–62, 66, 69, 76–82, 93, 113, 114, 116, 117]	38 (44.19%)	Low	0–70%	15–83 days
No pain during strengthening or stretching [13, 15, 20, 40, 42, 43, 46, 50, 53, 54, 58–60, 66, 70, 76, 117]	17 (19.77%)	Low	0–20.7%	15–51 days
External load progression criteria	18 (20.93%)			
Progression and control of match exposure [10, 21, 22, 55, 67, 71, 83, 84, 114]	9 (10.47%)	Very low	0%	22.42 days
At least 1–5 full training sessions before return to competition [21, 52, 56, 57]	4 (4.65%)	Very low	9.3–20%	15.5–30 days
Similar GPS profile in training sessions [13, 20, 21, 23, 76, 116, 118]	7 (8.14%)	Very low	0%	22.42–29 days
Jumping-derived criteria	9 (10.47%)			
Similar jumping kinetics [85, 110]	2 (2.33%)	Very low	Data not available	Data not available
Similar jumping performance [15, 42, 44, 51, 70, 86, 87]	7 (8.14%)	Low	0–70%	22.2–37.4 days
Strength criteria	46 (53.49%)			
Similar hip strength [15, 76, 85, 88]	4 (4.65%)	Very low	Data not available	Data not available
Similar knee flexors/extensors strength [13, 15, 20–23, 40, 41, 46, 48, 50, 51, 53, 56–59, 63, 64, 66, 69, 70, 72, 77, 80, 81, 85, 89–97, 109, 112, 114, 116, 117]	42 (48.84%)	Low	0–23.07%	21–51 days
Similar angle of isokinetic peak torque [58, 72]	2 (2.33%)	Very low	Data not available	Data not available
Similar muscle strength endurance [15, 68, 98, 114]	4 (4.65%)	Very low	Data not available	Data not available
Range of motion criteria	17 (19.77%)			
Similar active knee extension test [21, 40, 42, 43, 52, 70, 77]	7 (8.14%)	Moderate	0–11.11%	15–42.5 days
Similar passive straight leg raise test [13, 20, 21, 42, 43, 51, 52]	7 (8.14%)	Moderate	0–11.11%	15–23.82
Similar performance in Askling-H or ASLR test [15, 20, 42, 114]	4 (4.65%)	Moderate	0–25%	23.09–25.5 days
Similar unspecified range of motion [13, 23, 58, 66, 91, 113]	6 (6.98%)	Very low	15.7%	22.2–43 days
Biomechanics criteria	3 (3.49%)			
Symmetry in kicking kinematics [99]	1 (1.16%)	Very low	Data not available	Data not available
Adequate lumbopelvic control [21]	1 (1.16%)	Very low	Data not available	Data not available
Symmetry in sprinting biomechanics [100]	1 (1.16%)	Very low	Data not available	Data not available
Muscle activation criteria	7 (8.14%)			
Similar knee flexors activation [86, 95, 101, 102, 110, 112]	6 (6.98%)	Very low	Data not available	Data not available
Similar gluteal activation [102, 103]	2 (2.33%)	Very low	Data not available	Data not available
Adequate trunk muscles activation [103]	1 (1.16%)	Very low	Data not available	Data not available
Muscle imaging criteria	14 (16.28%)			
Similar muscle architecture [89, 94, 111]	3 (3.49%)	Very low	Data not available	Data not available
Maturity of the scar tissue in MRI or ultrasound [20, 23, 47, 51, 65, 66, 73, 92, 104, 105, 114]	11 (12.79%)	Very low	12–15.7%	22.2–36.7 days
Subjective perceived effort criteria	1 (1.16%)			
Lower rate of perceived exertion after repeated sprint tasks [87]	1 (1.16%)	Very low	Data not available	Data not available
Psychology criteria	13 (15.12%)			
Subjective readiness [13, 20–23, 41, 43, 46, 51, 76, 78, 113, 115]	13 (15.12%)	Moderate	7.14–23.07%	15–33 days
Healing criteria	4 (4.65%)			
Adequate time of recovery according to the degree of the injury [45, 61, 105, 114]	4 (4.65%)	Very low	Data not available	Data not available
Agility and soccer-specific ability criteria	8 (9.30%)			
Similar CoD performance [20, 58]	2 (2.33%)	Very low	Data not available	Data not available
Similar sprinting performance [13, 21, 74, 75, 106, 107]	6 (6.98%)	Very low	Data not available	Data not available
Muscle tissue contractile and sensorial properties criteria	3 (3.49%)			
Recover tissue stiffness [82, 108]	2 (2.33%)	Very low	Data not available	Data not available
Similar proprioceptive capacity [86, 108]	2 (2.33%)	Very low	Data not available	Data not available
Fitness criteria	1 (1.16%)			
Recover preinjury aerobic/anaerobic performance [21]	1 (1.16%)	Very low	Data not available	Data not available

The term “similar” here refers to “similar to preinjury levels” and “similar to contralateral limb”

*GPS* global positioning system, *ASLR* active single-leg raise, *MRI* magnetic resonance imaging, *CoD* change of direction

Specific domains for the no pain criteria following hamstring injuries were not having pain at palpation of the posterior thigh [13, 15, 20, 40–43, 52, 66, 77, 113, 114]; in soccer-specific actions such as sprinting [22, 42–45, 52–54, 66, 77, 78, 117], jumping [42, 45], or drills at the end of the rehabilitation program [13, 20, 21, 23, 46–49, 51, 53–56, 59–62, 66, 69, 76, 78–82, 93, 114, 116, 117]; and during strengthening exercises focused on the posterior chain [13, 15, 20, 40, 42, 43, 46, 50, 52–54, 58–60, 66, 70, 76, 117] such as bridge [53, 66], resisted knee flexion [13, 20, 40, 42, 43, 58, 60, 70], or squat [53, 66]. Domains for external load progression involved assessing progression of match exposure, including the number of minutes played in both training and competition [10, 21, 22, 55, 67, 71, 83, 84, 114]. This also included completing one to five full training sessions with the rest of the team [21, 52, 56, 57] and presenting a global positioning system (GPS) profile (external load) similar to the microcycles prior to injury [13, 20–23, 76, 116, 118]. Jumping kinetic and kinematic domains were further divided into kinetic-derived criteria. This included evaluating similarity of the jumping profile measured through force plates to preinjury and contralateral limb conditions [85, 110]. Additionally, assessing similar performance in jumping across different planes of movement [15, 42, 44, 51, 70, 86, 87] was considered. Domains based on strength criteria were created for hip strength [15, 76, 85, 88], knee flexor/extensor strength [13, 15, 20–23, 40, 41, 46, 48, 50, 51, 53, 56–59, 63, 64, 66, 69, 70, 72, 77, 80, 81, 85, 89–94, 96, 97, 111, 114, 116, 117, 157], angle of peak torque in isokinetic assessment [58, 72], and similar hamstring fatigability [15, 68, 98, 114]. Range of motion domains were based on active knee extension [21, 40, 42, 43, 52, 70, 77], passive straight leg raise [13, 20, 21, 42, 43, 51, 52], Askling-*H* and active straight leg raise [15, 20, 42, 114] tests, and unspecified range of motion tests [13, 23, 58, 66, 91, 113]. Domains related to biomechanics were interlimb symmetry in kicking kinematic analysis [99], proper lumbopelvic control during basic movement patterns in soccer [21], and symmetry between limbs during sprinting kinematic analysis [100]. Criteria for muscle activation included assessing activation of knee flexors [86, 101, 102, 110, 111, 157] and glutes [102, 103] and ensuring good activation of trunk muscles [103]. Muscle imaging domains were established to evaluate similar muscle architecture, considering factors such as muscle thickness, fascicle length, and pennation angle [89, 94, 111]. Additionally, signals of maturity in the scar tissue, observed through magnetic resonance imaging (MRI) or ultrasound just before RTP [20, 23, 47, 51, 65, 66, 73, 92, 104, 105, 114], were considered. For the rate of perceived exertion, the lower subjective perception of effort after a repeated sprint ability task [87] was considered as the only domain. For psychology, the only domain was that the player felt ready to RTP [13, 20–23, 41, 43, 46, 51, 76, 78, 113, 115] and especially sprinting [41, 43], without fear of reinjury [20, 22, 46] and with a perception of similar hamstring function between limbs [78]. For healing criteria, adequate recovery (i.e., respecting healing process) according to the extent and degree of injury (e.g., based on initial injury severity grading) was necessary for RTP [45, 61, 105, 114]. In the assessment of soccer-specific abilities, domains were established to evaluate similar performance in sprinting [13, 21, 74, 75, 106, 107] and changing direction at multiple angles [20, 58]. Muscle tissue properties were assessed based on recovery of hamstring tissue stiffness [82, 108] and similar proprioceptive capacity [86, 108]. Finally, the domain for fitness criteria focused on recovering preinjury aerobic and anaerobic performance [21]. An overview of results classified by RTP criteria, domain, and type of study is presented in Table 2.

**Table 2:** Summary of results of the included studies filtered by return-to-play criteria, domain, and type (i.e., design) of study following hamstring injuries

RTP criteria	Specific domain	Randomized controlled trials	Longitudinal cohort studies	Prospective	Case study
Pain	No pain at palpation [13, 15, 20, 40–43, 52, 66, 77, 113, 114]	No pain [15, 40–51]	No pain [52–56, 58–62, 66, 116, 117]	No pain [15, 41–45, 48–51, 53, 66, 69, 93, 117]	No pain during resisted knee flexion and during full range of motion [70, 76]
	No pain during soccer-specific actions [13, 15, 20–23, 42–49, 51–56, 59–62, 66, 69, 76–82, 93, 113, 114, 116, 117]				
	No pain during strengthening or stretching [13, 15, 20, 40, 42, 43, 46, 50, 53, 54, 58–60, 66, 70, 76, 117]				
External load progression	Progression and control of match exposure [10, 21, 22, 55, 67, 71, 83, 84, 114]		Recover GPS microcycle training demands [10, 52, 55–57, 116, 118]	Progression of match exposure [10, 55, 67]	Progression of match exposure and achievement of preinjury running metrics [71, 76]
	At least 1–5 full training sessions before return to competition [21, 52, 56, 57]				
	Similar GPS profile in training sessions [13, 20, 21, 23, 76, 116, 118]				
Jumping kinetics and kinematics	Similar jumping kinetics [85, 110]	Recover single, triple and crossover hop performance [15, 42, 44, 51]		Recover single, triple and crossover hop performance [15, 42, 44, 51]	Similar single leg hop [70]
	Similar jumping performance [15, 42, 44, 51, 70, 86, 87]				
Strength	Similar hip strength [15, 76, 85, 88]	Similar strength and fatigability [15, 40, 41, 46, 48, 50, 51]	Similar knee strength and angle of peak torque [53, 56–59, 63, 64, 66, 116, 117]	Similar strength and fatigability [15, 41, 48, 50, 51, 53, 63, 66, 68, 69, 93, 97, 117]	Bilateral isometric asymmetry < 10%, isokinetic concentric H:Q ratio > 0.66 [70, 72, 76]
	Similar knee flexors/extensors strength [13, 15, 20–23, 40, 41, 46, 48, 50, 51, 53, 56–59, 63, 64, 66, 69, 70, 72, 77, 80, 81, 85, 89–97, 109, 112, 114, 116, 117]				
	Similar angle of isokinetic peak torque [58, 72]				
	Similar muscle strength endurance [15, 68, 98, 114]				
Range of motion	Similar active knee extension test [21, 40, 42, 43, 52, 70, 77]	Similar active and passive hamstrings flexibility [15, 40, 42, 43, 51]	Similar hip range of motion [52, 58, 66]	Similar hip and knee range of motion [15, 42, 43, 66]	Restore 90–90 hamstrings flexibility test [70]
	Similar passive straight leg raise test [13, 20, 21, 42, 43, 51, 52]				
	Similar performance in Askling-H or ASLR test [15, 20, 42, 114]				
	Similar unspecified range of motion [13, 23, 58, 66, 91, 113]				
Biomechanics	Symmetry in kicking kinematics [99]				
	Adequate lumbopelvic control [21]				
	Symmetry in sprinting biomechanics [100]				
Muscle activation	Similar knee flexors activation [86, 95, 101, 102, 110, 112]				
	Similar gluteal activation [102, 103]				
	Adequate trunk muscles activation [103]				
Muscle imaging	Similar muscle architecture [89, 94, 111]	Absence of swelling in the injured site [47, 51]	MRI-confirmed healed tissue [65, 66]	MRI or ultrasound confirmed healed tissue [51, 66]	Complete healing of the muscle and tendon in MRI [73]
	Maturity of the scar tissue in MRI or ultrasound [20, 23, 47, 51, 65, 66, 73, 92, 104, 105, 114]				
Rate of perceived exertion	Lower rate of perceived exertion [87]				
Psychology	Subjective readiness [13, 20–23, 41, 43, 46, 51, 76, 78, 113, 115]	Subjective readiness [41, 43, 46, 51]		No apprehension during sprinting [41, 43, 51]	Positive player feedback after soccer-specific drills [76]
Healing	Adequate time of recovery according to the degree of the injury [45, 61, 105, 114]	Respect biological healing in myotendinous junction injuries [45]	Adequate time of healing [61]	Respect biological healing in musculotendinous junction injuries [45]	
Agility/soccer-specific ability	Similar CoD performance [20, 58]		Replicate specific movements at competition speed [58]		Similar horizontal force–velocity profile [74, 75]
	Similar sprinting performance [13, 21, 74, 75, 106, 107]				
Muscle tissue contractile and sensorial properties	Recover tissue stiffness [82, 108]				
	Similar proprioceptive capacity [86, 108]				
Fitness	Recover preinjury aerobic/anaerobic performance [21]				

Sentences accompanied by numbers in brackets summarize the evidence for a specific criterion and the studies reporting it, respectively

*GPS* global positioning system, *ASLR* active single-leg raise, *MRI* magnetic resonance imaging, *CoD* change of direction. The term “similar” here refers to similar to preinjury levels and similar to the contralateral limb

Available evidence for RTP criteria after adductors injury in soccer was classified as: (1) absence of pain, (2) biomechanics, (3) soccer-specific ability, (4) external load progression, (5) strength, (6) muscle imaging, (7) subjective outcomes, (8) range of motion, and (9) healing (Table 3). The absence of pain domains were absence of clinical symptoms [20, 119, 129, 132, 133]; no pain at adductor palpation [17, 20, 121, 122]; no pain during resisted contractions such as isometric hip adduction in outer-range abduction [17, 121, 122, 128], Copenhagen adduction exercise [17, 121, 122], resisted concentric/eccentric contractions [20], or strength training [125]; no pain during maximal passive stretching [17, 20, 121, 122]; no pain during on-field and agility tasks such as linear sprinting [17, 121, 122], jogging [125], agility T test [17, 121, 122], and full soccer training sessions [47, 61, 123, 128, 130, 134]; and absence of pain in pubic stress test [20]. Biomechanics-based domains were similar preinjury anterior and total pelvic tilt [120, 126] and having good quality of movement, especially in fundamental movement patterns [133]. Soccer-specific ability domains were similar preinjury performance on changing of direction tests [17, 20, 121, 122, 133] such as the Illinois agility test [17, 121, 122], spider test [17, 121, 122], carioca test [20], or planned and unplanned change of directions [133]; similar performance on technical–tactical tasks [17, 20, 121, 122, 133] such as straight passes progressing distance [17, 121, 122], crosses standing and running [17, 121, 122], shooting scenarios [17, 121, 122], one versus one drills [17, 121, 122], and qualitatively similar performance in full training sessions [20, 133]; and similar preinjury jumping performance after a repeated sprint ability task [87]. External load progression domains were completion of one full team training session [17, 47, 121, 122], < 10% preinjury difference in maximal speed, and high-speed running and sprints with > 70% game load [134] within 7–10 days after returning to training [20]. Strength domains were similar preinjury and contralateral hip adduction strength [20, 92, 123, 126–128, 133] with < 10% [123] to 15% [128] asymmetry; similar preinjury and contralateral abduction strength [126, 127]; similar hip adduction/abduction ratio [127, 128]; similar preinjury and contralateral eccentric adduction strength [124, 133]; similar hip flexion strength compared with preinjury, contralateral limb, and normative data for soccer players [126]; similar hip extension strength compared with preinjury, contralateral limb, and normative data for soccer players [126]; similar preinjury and contralateral isokinetic strength [92, 126, 129] at 60°/s [126] and 240°/s [126]; and similar preinjury and contralateral isokinetic hamstring/quadriceps ratio [126]. Muscle imaging domains were maturity of the scar in magnetic resonance imaging (MRI) or ultrasound [20, 92, 134], ensuring correct alignment of muscle fibers without edema [134], hyperintensity zone reduction of ≥ 70%, assessment of low signal intensity as a measure of fibrotic tissue [20], and absence of swelling [47, 134]. Subjective outcome domains were subjective readiness [20, 132, 133]; levels of anxiety [20], apprehension [20], fear of failure [20], and fear of reinjury [20]; Lower Extremity Functional Score [132] or Global Rating of Charge [132]; adequate and improved score in the Copenhagen Hip and Groin Outcome Score (HAGOS) scale [128]; and lower rate of perceived exertion after a repeated sprint ability task [87]. Range of motion criteria were classified in one domain of similar preinjury and contralateral hip abduction [20]. Healing criteria were classified in one domain of adequate recovery according to the degree of the diagnosed injury [61, 131]. An overview of adductors injury results classified by RTP criteria, domain, and type of study is presented in Table 3.

**Table 3:** Summary of results of included studies filtered by return-to-play criteria following adductors injury, domain, and type (i.e., design) of study

RTP criteria	Number of studies reporting this criterion (*n* (%))	Certainty of evidence	Randomized controlled trials	Longitudinal cohort studies	Prospective	Case study
Pain criteria	15 (68.18%)		No pain during strength exercises and team training sessions [47, 125]	Absence of pain [17, 61, 121, 122, 129, 130, 134]	Absence of pain [47, 122, 129, 134]	No pain without aggravation of symptoms the following days after soccer training [119, 123, 132]
Absence of clinical symptoms [20, 119, 129, 132, 133]	5 (22.73%)	Very low				
No pain at palpation [17, 20, 121, 122]	4 (18.18%)	Very low				
No pain during resisted contraction [17, 20, 121, 122, 125, 128]	6 (27.27%)	Moderate				
No pain during passive stretching [17, 20, 121, 122]	4 (18.18%)	Very low				
No pain during on-field/agility tasks [17, 20, 47, 61, 121–123, 125, 128, 130, 134]	11 (50%)	Moderate				
No pain in pubic stress test [20]	1 (4.55%)	Very low				
Biomechanics criteria	3 (13.63%)					
Similar preinjury anterior and total pelvic tilt [120, 126]	2 (9.09%)	Very low				
Good quality of movement [133]	1 (4.54%)	Very low				
Soccer-specific abilities	6 (27.27%)			100% intensity on Illinois and spider test, with completion of match situations [17, 121, 122]	Similar Illinois agility and spider test with completion of match situations [121]	
Similar change of direction performance [17, 20, 121, 122, 133]	5 (22.73%)	Very low				
Similar performance on technical–tactical tasks [17, 121, 122]	3 (13.64%)	Very low				
Similar jumping performance after repeated sprint ability [87]	1 (4.54%)	Very low				
External load progression	6 (27.27%)		Completion of team training session demands [47]	Complete team training sessions with < 10% difference in GPS parameters [17, 121, 122]	Complete team training sessions with < 10% difference in GPS parameters [122, 134]	
Completion of one full team training session [17, 47, 121, 122]	4 (18.18%)	Moderate				
Less than 10% difference of GPS-based performance in training session [20, 134]	2 (9.09%)	Very low				
Strength	8 (36.36%)			Similar isokinetic adduction and abduction strength [92, 129]	Similar isokinetic adduction and abduction strength [92, 129]	Asymmetry of < 10% between injured and uninjured limbs in hip adduction strength [123]
Similar hip adduction strength [123, 126–128]	4 (18.18%)	Very low				
Similar hip abduction strength [126, 127]	2 (9.09%)	Very low				
Similar hip adduction/abduction ratio [127, 128]	2 (9.09%)	Very low				
Similar eccentric hip adduction strength [124, 133]	2 (9.09%)	Very low				
Similar hip flexion strength [126]	1 (4.54%)	Very low				
Similar hip extension strength [126]	1 (4.54%)	Very low				
Similar isokinetic strength [92, 126, 129]	3 (13.64%)	Very low				
Similar isokinetic hamstrings/quadriceps ratio [126]	1 (4.55%)	Very low				
Muscle imaging criteria	4 (18.18%)		Absence of swelling in the injured site [47]	Correct alignment of muscle fibers and no evidence of edema [92, 134]	Correct alignment of muscle fibers with no swelling [47, 92, 134]	
Maturity of the scar in MRI or ultrasound [20, 92, 134]	3 (13.64%)	Very low				
Absence of swelling [47, 134]	2 (9.09%)	Low				
Subjective outcomes	5 (22.73%)					Improvement in LEFS and Global Rating of Charge [132]
Subjective readiness [20, 132, 133]	3 (13.64%)	Very low				
Adequate and improved HAGOS scale [128]	1 (4.55%)	Very low				
Lower rate of perceived exertion after repeated sprint ability [87]	1 (4.55%)	Very low				
Range of motion criteria	1 (4.55%)					
Similar hip abduction range of motion [20]	1 (4.55%)	Very low				
Healing criteria	2 (9.09%)			Adequate rehabilitation time according to the extent of the injury [61, 131]		
Adequate time of recovery according to the degree of the injury [61, 131]	2 (9.09%)	Very low				

*RTP* return-to-play, *RCT* randomized controlled trial, *GPS* global positioning system, *MRI* magnetic resonance imaging, *HAGOS* Copenhagen Hip and Groin Outcome Score, *CMJ* countermovement jump, *RSA* repeated sprint ability, *LEFS* Lower Extremity Functional Scale

Available evidence for RTP criteria after quadriceps injury was classified as: (1) absence of pain, (2) soccer-specific ability, (3) external load progression, (4) strength, (5) muscle imaging, (6) subjective outcomes, (7) range of motion, (8) healing, and (9) muscle activation (Table 4). Absence of pain domains were absence of clinical symptoms [20, 45, 135, 138–140], no pain at palpation of the injured area in the quadriceps [20], no pain during resisted contraction (i.e., knee extension and hip flexion) [20], no pain during passive stretching [20], no pain during on-field and agility tasks [11, 45, 47, 61, 137] such as multidirectional skills and acceleration/deceleration [11], no pain during and after repeated sprint ability tasks [45], no pain in single-leg jump [45], and no pain kicking [11]. Soccer-specific ability domains were based on similar change of direction performance in the Illinois agility test and braking test [20] and similar performance on technical–tactical tasks such as individual and team-specific drills [11, 140]. External load progression domains were the completion of at least one full team training session [47, 61], and < 10% difference of GPS-based performance in training sessions [11, 20]. Strength-related domains were similar knee extension strength [20, 92, 137], with one study fixing the 95% symmetry threshold [137], and similar isokinetic strength [92]. Muscle imaging domains were the maturity of the scar on MRI or ultrasound [20, 92], checking completion of full tissue [92], hyperintensity zone reduction of ≥ 70% [20], and absence of swelling [47, 137, 139]. Subjective outcome criteria were classified in one domain of subjective readiness [20]. Range of motion criteria were classified in one domain of similar active and passive hip and knee range of motion [20, 137, 139, 140]. Healing criteria were classified in one domain of adequate time of recovery according to degree of the injury [45, 61]. Muscle activation criteria were classified in one domain of similar rectus femoris activation in the proximal portion [136, 141]. An overview of quadriceps injury results classified by RTP criteria, domain, and type of study is presented in Table 4.

**Table 4:** Summary of results of included studies filtered by return-to-play criteria following quadriceps injury, domain, and type (i.e., design) of study

RTP criteria	Number of studies reporting this criterion (*n* (%))	Certainty of evidence	Randomized controlled trials	Longitudinal cohort studies	Prospective	Case study
Pain criteria	10 (76.92%)		Pain-free during on-field rehabilitation [45, 47]	No tenderness in the injured area and no pain kicking [11, 61, 135, 140]	No pain during full team training sessions [11, 45, 47]	Absence of pain or impairments in on-field training [137–139]
Absence of clinical symptoms [20, 45, 135, 138–140]	6 (46.15%)	Low				
No pain at palpation [20]	1 (7.69%)	Very low				
No pain during resisted contraction [20]	1 (7.69%)	Very low				
No pain during passive stretching [20]	1 (7.69%)	Very low				
No pain during on-field/agility tasks [11, 45, 47, 61, 137]	5 (38.46%)	Low				
No pain during and after repeated sprint ability [45]	1 (7.69%)	Low				
No pain in single-leg jump [45]	1 (7.69%)	Low				
No pain kicking [11]	1 (7.69%)	Very low				
Soccer-specific abilities	3 (23.08%)			Maximal output in multidirectional and on-field skills [11, 140]	Completion of all programmed individual sport-specific drills [11]	
Similar change of direction performance [20]	1 (7.69%)	Very low				
Similar performance on technical–tactical tasks [11, 20, 140]	3 (23.08%)	Very low				
External load progression	4 (30.77%)		To participate in team training sessions without pain [47]	Complete team training sessions with < 10% difference in GPS parameters [11, 61]	Completion of the full practice with > 90% maximal speed, HSR and sprint variables [11, 47]	
Completion of one full team training session [47, 61]	2 (15.38%)	Low				
Less than 10% difference of GPS-based performance in training session [11, 20]	2 (15.38%)	Very low				
Strength	3 (23.08%)			Correction of the strength imbalances [92]	Correction of the strength imbalances [92]	Knee extension strength at least 95% of the uninjured limb [137]
Similar knee extension strength [20, 92, 137]	3 (23.08%)	Very low				
Similar isokinetic hamstrings and quadriceps strength [92]	1 (7.69%)	Very low				
Muscle imaging criteria	5 (38.46%)		Absence of swelling in the injured site [47]	Completion of the healing process through imaging validation [92]	Healed muscle on imaging without swelling [47, 92]	Absence of edema and injury signs on MRI and ultrasound [137, 139]
Maturity of the scar in MRI or ultrasound [20, 92]	2 (15.38%)	Very low				
Absence of swelling [20, 47, 137, 139]	4 (30.77%)	Low				
Subjective outcomes	1 (7.69%)					
Subjective readiness [20]	1 (7.69%)	Very low				
Range of motion criteria	3 (23.08%)			Full knee and hip range of motion [140]		Full range of motion, with at least 130º knee flexion [137, 139]
Restore hip and knee range of motion with at least 130° knee flexion [20, 139, 140]	3 (23.08%)	Very low				
Healing criteria	2 (15.38%)		Adequate healing in musculo-tendinous injuries [45]	Adequate time to return to play according to the extent of the injury [61]	Adequate healing in musculo-tendinous junction injuries [45]	
Adequate time of recovery according to the degree of the injury [45, 61]	2 (15.38%)	Very low				
Muscle activation criteria	2 (15.38%)					
Similar rectus femoris activation in proximal portion [136, 141]	2 (15.38%)	Very low				

*RTP* return-to-play, *RCT* randomized controlled trial, *GPS* global positioning system, *MRI* magnetic resonance imaging, *HSR* high-speed running, *EMG* electromyography

Available evidence for RTP criteria following calf injury was classified as: (1) absence of pain, (2) soccer-specific ability, (3) external load progression, (4) strength, (5) muscle imaging, (6) subjective outcomes, (7) range of motion, and (8) healing (Table 5). Specific domains for the absence of pain criteria were absence of clinical signs and symptoms [14, 18, 20, 143], absence of pain at palpation in the injured area [14, 20], no pain during resisted contraction (i.e., plantar flexion) [20], absence of pain during passive stretching of the calf [20], no pain during soccer-specific on-field and agility tasks [20, 47, 61], and absence of pain during ambulation [142]. Regarding soccer-specific abilities, specific domains were similar change of direction performance [14, 20] such as the Illinois agility test [20], similar performance on technical–tactical tasks [14, 20], similar drop jump test [20], and similar vertical and horizontal hop [14]. External load progression domains were based on similar GPS profiles, especially in total volume, volume of high-speed running, and acceleration or deceleration variables [14, 143] with a < 10% difference of GPS-based performance in training session [20] and completing at least one full team training session [20, 47, 61]. Strength-derived domains were similar performance in calf raise test [14, 20, 143] with a threshold of < 10% asymmetry [14], similar plantar flexion strength [20], similar synchro plates test [20], and ≥ 30 repetitions on the single-leg calf raise test [14]. Muscle imaging domains were the maturity of the scar on MRI or ultrasound [20, 145], similar shear wave elastography [145], and absence of swelling [47]. Subjective outcomes were classified with one domain categorized as subjective readiness of the player [20]. Range of motion criteria also showed one domain classified as similar ankle flexibility test [20, 143]. Healing-related criteria were classified in one domain of adequate recovery according to degree of the injury [18, 61, 144]. An overview of calf injury results classified by RTP criteria, domain, and type of study is presented in Table 5.

**Table 5:** Summary of results of included studies filtered by return-to-play criteria following calf injury, domain, and type (i.e., design) of study

RTP criteria	Number of studies reporting this criterion (*n* (%))	Certainty of evidence	Randomized controlled trials	Longitudinal cohort studies	Prospective	Cross-sectional	Case study	Expert consensus
Pain criteria	7 (77.78%)		To participate in team training sessions without pain [47]	No tenderness in the injured area and no pain kicking [18, 61, 142]	Absence of pain during ambulation and soccer training [18, 47, 142]		Absence of daily clinical signs and symptoms [143]	Absence of pain [14, 20]
Absence of clinical symptoms [14, 18, 20, 143]	4 (44.44%)	Very low						
No pain at palpation [14, 20]	2 (22.22%)	Very low						
No pain during resisted contraction [20]	1 (11.11%)	Very low						
No pain during passive stretching [20]	1 (11.11%)	Very low						
No pain during on-field/agility tasks [20, 47, 61]	3 (33.33%)	Low						
No pain during ambulation [142]	1 (11.11%)	Very low						
Soccer-specific abilities	2 (22.22%)							Similar on-field performance with emphasis on CoD and jumping performance [14, 20]
Similar change of direction performance [14, 20]	2 (22.22%)	Very low						
Similar performance on technical–tactical tasks [14, 20]	2 (22.22%)	Very low						
Similar drop jump test [20]	1 (11.11%)	Very low						
Similar vertical and horizontal hop [14]	1 (11.11%)	Very low						
External load progression	5 (55.55%)		To participate in team training sessions without pain [47]	Successful completion of scrimmages [61]	To participate in team training sessions without pain [47]		Same profile in total distance, speed, high intensity running, accelerations and decelerations [143]	Similar preinjury full team training GPS external load profile [14, 20]
Similar GPS profile [14, 143]	2 (22.22%)	Very low						
Less than 10% difference of GPS-based performance in training session [20]	1 (11.11%)	Very low						
Completion of one full training session [14, 47, 61]	3 (33.33%)	Low						
Strength	3 (33.33%)						Similar preinjury and contralateral calf raise test [143]	Similar strength profile of the player [14, 20]
Similar calf raise test [14, 20, 143]	3 (33.33%)	Very low						
Similar plantar flexion strength [20]	1 (11.11%)	Very low						
Similar synchro plates test [20]	1 (11.11%)	Very low						
≥ 30 repetitions on single leg calf raise test [14]	1 (11.11%)	Very low						
Muscle imaging criteria	3 (33.33%)		Absence of swelling in the injured site [47]		Absence of swelling in the injured site [47]	Large scar thickness in ultrasound and similar Shear Wave Elastography [145]		Hyperintensity reduction of 70% and check low signal intensity [14]
Maturity of the scar in MRI or ultrasound [20, 145]	2 (22.22%)	Very low						
Similar shear wave elastography [145]	1 (11.11%)	Very low						
Absence of swelling [47]	1 (11.11%)	Low						
Subjective outcomes	1 (11.11%)							Low levels of anxiety, apprehension, fear of failure and/or fear of reinjury [20]
Subjective readiness [20]	1 (11.11%)	Very low						
Range of motion criteria	2 (22.22%)						Full range of motion in dorsiflexion lunge test [143]	Similar ankle flexibility test [20]
Similar dorsiflexion [20, 143]	2 (22.22%)	Very low						
Healing criteria	3 (33.33%)			Respect biological healing process according to the extent of the injury [18]	Respect biological healing process [18]	Check the return to play in a normal interval for the grade of the injury [144]		
Adequate time of recovery according to the degree of the injury [18, 61, 144]	3 (33.33%)	Very low						

*RTP* return-to-play, *RCT* randomized controlled trial, *GPS* global positioning system, *MRI* magnetic resonance imaging, *CoD* change of direction

RTP criteria implemented in studies in which the affected muscle group was not specified or established criteria for any lower limb injury were classified as general lower limb criteria. Available evidence for general lower limb RTP criteria was classified as: (1) absence of pain, (2) soccer-specific ability, (3) external load progression, (4) jumping performance, (5) muscle imaging, (6) subjective outcomes, (7) range of motion, (8) anthropometry, and (9) movement quality (Table 6). Absence of pain domains were absence of clinical symptoms [147], absence of pain during resisted contraction [154], no pain during passive stretching of the affected muscle [154], and no pain during on-field or agility tasks [146, 154, 155] such as sprinting [155]. Soccer-specific ability domains were similar change of direction performance [146] such as the Barrow test or shuttle 8 × 5 m test with < 10% difference [146], and similar performance on technical–tactical tasks [9, 146, 148, 149, 156]. External load progression domains were the completion of four full team training sessions [150, 155], similar GPS-based performance in training sessions especially similar preinjury competition total distance covered sprinting or maximal running speed [149], and similar sprinting demands [9, 149]. Jumping performance domains were similar countermovement jump performance [146], similar unilateral jump [146], similar triple hop [146], and similar jumping kinetics [153], specifically reduced asymmetry in concentric (i.e., concentric impulse 100 ms, concentric peak force, and force at zero velocity) and eccentric (i.e., eccentric deceleration rate of force development, eccentric peak force, and eccentric/concentric force ratio) countermovement jump variables [153]. Muscle imaging domains were maturity of the scar in MRI or ultrasound [151, 152] and absence of swelling [146, 151]. Psychological domains were subjective readiness [146] and low score on POMS questionnaire [146]. Range of motion criteria were classified in one domain of similar unspecified range of motion [154]. Anthropometry-related criteria were classified in one domain of < 0.5% change in fat [146]. Movement quality criteria were classified in one domain of < 2 cm asymmetry in Y balance test [146]. The number of studies implementing each RTP criterion, categorized by domain and study design, is presented in Table 6.

**Table 6:** Summary of results of included studies filtered by return-to-play criteria following general lower limb muscle injury, domain, and type (i.e., design) of study

RTP criteria	Number of studies reporting this criterion (*n* (%))	Certainty of evidence	Randomized controlled trials	Longitudinal cohort studies	Prospective	Case study
Pain criteria	4 (33.33%)			No pain sprinting and RTP with medical staff assessment [49, 146, 147]	No pain during on-field tasks with special focus on sprint [49, 146]	
Absence of clinical symptoms [147]	1 (8.3%)	Very low				
No pain during resisted contraction [154]	1 (8.3%)	Very low				
No pain during passive stretching [154]	1 (8.3%)	Very low				
No pain during on-field/agility tasks [146, 154, 155]	3 (25%)	Very low				
Soccer-specific abilities	5 (41.67%)			Similar Barrow and shuttle 8 × 5 m tests with similar functional performance [146, 149, 156]	Similar Barrow and shuttle 8 × 5 m tests with similar functional performance [146]	
Similar change of direction performance [146]	1 (8.3%)	Very low				
Similar performance on technical–tactical tasks [9, 146, 148, 149, 156]	5 (41.67%)	Very low				
External load progression	5 (41.67%)			Participate in team training sessions with similar GPS-based performance [149, 150, 155]	Complete 4 team training sessions with 20 min of conventional soccer match [150, 155]	
Completion of four full team training sessions [150, 155]	2 (16.67%)	Very low				
Similar GPS-based performance in training session [149]	1 (8.3%)	Very low				
Similar sprinting demands [9, 149]	2 (16.67%)	Very low				
Jumping performance	2 (16.67%)			< 3 cm difference in CMJ and > 90% symmetry in unilateral and triple hop [146]	< 3 cm difference in CMJ and > 90% symmetry in unilateral and triple hop [146]	
Similar CMJ performance [146]	1 (8.3%)	Very low				
Similar unilateral jump [146]	1 (8.3%)	Very low				
Similar triple hop [146]	1 (8.3%)	Very low				
Similar jumping kinetics [153]	1 (8.3%)	Very low				
Muscle imaging criteria	2 (16.67%)			Reduced signal intensity in MRI without swelling [146]	Reduced signal intensity in MRI without swelling [146]	
Maturity of the scar in MRI or ultrasound [151]	1 (8.3%)	Very low				
Absence of swelling [146, 151]	2 (16.67%)	Very low				
Psychology	1 (8.3%)			Low POMS depression and fatigue, high POMS vitality and perceived readiness [146]	Low POMS depression and fatigue, high POMS vitality and perceived readiness [146]	
Subjective readiness [146]	1 (8.3%)	Very low				
Low score in POMS questionnaire [146]	1 (8.3%)	Very low				
Range of motion criteria	1 (8.3%)					
Similar unspecified range of motion [154]	1 (8.3%)	Very low				
Anthropometry	1 (8.3%)			< 0.5% in fat mass [146]	< 0.5% in fat mass [146]	
Changes < 0.5% in fat mass [146]	1 (8.3%)	Very low				
Movement quality	1 (8.3%)			< 2 cm asymmetry in Y balance test [146]	< 2 cm asymmetry in Y balance test [146]	
< 2 cm asymmetry in Y balance test [146]	1 (8.3%)	Very low				

*RTP* return-to-play, *RCT* randomized controlled trial, *GPS* global positioning system, *MRI* magnetic resonance imaging

### Study Designs and Certainty of the Evidence for RTP Criteria

An overview of the certainty of evidence for each domain as well as ranges of reinjury rates and time to RTP ranges as key variables [15, 158] following hamstring injuries are presented in Table 1. Details on risk of bias assessment for each study and GRADE certainty of evidence assessment for each domain are in Supplementary File 4. This information characterizes how extensively each domain has been investigated and which types of study designs have implemented specific RTP criteria, but it does not evaluate the effectiveness of individual criteria for reducing reinjury risk or time to RTP.

RTP criteria following adductors injury had moderate to very low certainty of evidence on the basis of the study design and methodological quality of the studies implementing these criteria, which can be found in Table 3. Criteria for RTP after quadriceps injury had low to very low certainty of evidence, and these results are presented in Table 4. Calf injury RTP criteria had low to very low certainty of evidence, and this is presented in Table 5. General lower limb muscle injury RTP criteria had a very low certainty of evidence for all classified domains, presented in Table 6. Supplementary File 4 shows the risk of bias assessment for the included studies, as well as the detailed GRADE classification for all domains.

The sensitivity analysis excluding studies in which soccer players represented less than 50% of the sample showed that only the use of subjective readiness as a RTP criterion following hamstring injuries would be affected with the more comprehensive inclusion of studies, with its level of certainty downgraded from moderate to low. The certainty of evidence for all other hamstring-specific RTP criteria remained unchanged. For adductor, quadriceps, calf and general lower limb muscle injuries, the sensitivity analysis revealed that the certainty of evidence for all criteria remained stable, with no changes observed.

## Discussion

### Understanding the Role of RTP Criteria Following Muscle Injuries

The primary aim of this systematic review was to systematically evaluate the available evidence on return-to-play (RTP) criteria following lower limb muscle injuries in soccer players. Our findings revealed that for hamstring injuries, the most frequently cited RTP criteria were strength symmetry between knee flexors/extensors and the absence of pain during soccer-specific actions, while range of motion assessments and subjective readiness criteria were implemented in RCTs with high methodological quality. For adductor injuries, criteria implemented in high methodological RCTs were absence of pain during resisted contractions or on-field tasks and completion of at least one full team training session. In contrast, criteria for quadriceps and calf injuries were cited only in longitudinal cohort studies and were not evaluated in any RCTs, primarily focusing on pain assessments and completing full training sessions. Overall, this review highlights both the most consistently used RTP criteria and the domains implemented in the highest methodological quality evidence, while also underscoring the need for more high-quality research, particularly for quadriceps and calf injuries. This study provides valuable insights for practitioners making RTP decisions given the lack of previous classifications of the certainty of evidence for clearance tests following muscle injuries. Such lack of graded evidence can lead to inefficient goals in rehabilitation by failing to define a point the player should reach. In addition, knowing how many studies report each criterion, as well as their design, is essential for practitioners to understand the characteristics of the existing evidence on RTP criteria and to make informed decisions on the basis of their specific context.

Given that RTP criteria are intended to ensure both safety and performance upon return, the end of the rehabilitation process should also include an assessment of the player’s overall fitness status [15, 159]; in this context, RTP criteria play a fundamental role in assessing if the player is ready to RTP. A backward design approach has been proposed in rehabilitation, whereby planning begins with the competition demands that the player must ultimately meet [159]. Therefore, RTP criteria after muscle injuries can represent the first step when designing the rehabilitation program and should address the specific context of the player as well as the goals of rehabilitation. This study categorized evidence-based RTP criteria by classifying into different certainties of evidence and informing the existing evidence (i.e., number of studies) reporting each criterion. It thus is important to implement criteria for multidimensional player assessment that can quantify lingering deficits that increase reinjury risk [15, 20]. At the end of the injury rehabilitation process (i.e., RTP), it is crucial to assess factors with the highest number of studies reporting them, as well as assessing those tests implemented in high quality RCTs, thus providing multidimensional information about the player’s condition [15, 160]. This review can function as a reference for practitioners to achieve efficiency in terms of time, economic and spatial resources when selecting the criteria for clearance. Notably, criteria with the highest certainty of evidence (i.e., mostly used in intervention studies with large cohorts such as RCTs and longitudinal cohort studies) in this study do not necessitate extensive resources for evaluation, and criteria most cited in literature do not necessarily coincide with those domains that have been most extensively investigated in higher-quality study designs.

### Hamstring Injury RTP Criteria

Strength criteria were the most frequently reported in scientific literature after hamstring injuries in soccer players, followed by pain-related criteria. In this context, achieving similar knee flexor/extensor strength and the absence of pain during soccer-specific actions were the most cited benchmarks. However, range of motion and psychological criteria were implemented in high quality RCTs. Specifically, domains such as exhibiting similar performance in active knee extension tests, passive straight leg raises, and Askling-H/active straight leg raises when compared with preinjury and the contralateral limb, along with subjective readiness, were more often implemented in RCTs with high methodological quality than other domains.

Similar strength capacity (i.e., from preinjury data or compared with contralateral limb at RTP) in the knee flexors/extensors is the most cited criterion for assessing in RTP. In this context, knee extensor strength is mentioned only to emphasize the importance of preserving an adequate hamstring-to-quadriceps (H:Q) ratio. This ratio should be at least maintained, and ideally increased when baseline values are poor, to minimize the risk of reinjury. However, it is important to note that strength criteria showed a low certainty of evidence, since most of the best available evidence (i.e., RCTs) showed some concerns such as a high risk of bias (Supplementary File 4). Nonetheless, several studies have reported assessment of isokinetic knee flexor/extensor strength as well as similar hamstrings to quadriceps ratio both prior to and following injury to support RTS decision making [15, 40, 46, 48, 53, 56, 57, 63, 64, 66, 69, 72, 81, 91–97, 109, 116, 117]. Incorporating isokinetic assessment may not be cost-effective and time-efficient; therefore, we suggest its use when assessment devices (e.g., Biodex or Cybex) are readily accessible. One simple strength test is the single bridge test [13, 15], which could offer information about strength of the posterior chain as well as its fatigability, another important dimension to assess for clearance, since injured hamstrings have lower strength endurance capacity and thus constitute an important risk factor for subsequent injuries [82, 98, 161]. A common cutoff point used to assess single leg bridge is > 25 repetitions (i.e., until failure with 20° knee flexion, box measuring 60-cm high and full range of motion) [15], and it also is important to quantify asymmetry between limbs owing to its predictive value for hamstring injuries [161]. Further, it is advisable to conduct isometric contractions at various knee angles to gather data on the hamstrings’ maximal force generation during analytical actions, such as resisted knee flexion [20, 41, 50]. Another crucial aspect for evaluation, which can be considered a strength-related action, is jumping. Thus, it is essential to assess a single hop for height [42, 44, 70], single leg four crossover [44] (i.e., zig-zag hopping), single leg triple hop [15, 86], and squat jump or countermovement jump after a repeated sprint ability task [87]. Therefore, assessment of jumping performance should be included in RTP screening.

It is important to consider the mechanism of injury and individual characteristics to design an adequate RTP strength assessment, as recently highlighted by Bramah et al. [162]. Several studies have established the main mechanisms of injury for hamstring injuries [6, 163], highlighting injuries during running patterns for both male and female players and specifically overstretching [163] and high-intensity accelerations [164] for professional players. Crucial aspects such as the differences in mechanisms of injury between sexes, whether the injury was preceded by a direct contact or not, as well as the limb involved [163] should be considered for designing RTP assessment protocols, adding this information to our presented graded criteria and thus existing the possibility to adapt the reported criteria and tests in this review. Therefore, screening for clearance after an injury should be specific for each injured soccer player according to the demands of each position [165], mechanism of injury [162, 166], or individual biomechanical parameters [162]. For example, knee flexor strength assessment could be performed in lower limb joint positions (e.g., similar hip and knee angles of flexion and internal/external rotation) according to the injury mechanism. Assessments to determine RTP clearance thus should be based on the results presented in this review and then individually adapted.

Pain criteria showed to be the second most cited criteria for RTP following hamstring injuries [13, 15, 20–23, 42–49, 51–56, 59–62, 66, 69, 76–82, 93, 113, 114, 116, 117]. In addition, it showed a low certainty of evidence (i.e., implemented in low methodological RCTs or longitudinal cohort studies), which represents the second highest certainty of evidence in this review. Specifically, presenting no pain during soccer-specific actions represented the second most cited criterion for assessment in RTP. In addition, it constitutes the most specific assessment for pain, since the absence of pain at palpation [13, 15, 20, 40–43, 52, 66, 77, 113, 114] and the absence of pain during strength exercises or stretching [13, 15, 20, 40, 42, 43, 46, 50, 53, 54, 58–60, 66, 70, 76, 117] can offer information about the status of the muscle–tendon tissue in an analytical approach, but exposing the player to specific drills should be essential for asking to any discomfort. Specifically, as mentioned with strength assessment, it is crucial to expose the player to activities that may be associated with the mechanism of the injury [162]. In this context, if the player was injured during a high-intensity acceleration, the main mechanism for male professional soccer players as recently reported [164], it will be necessary to ask for pain in this injury-inciting situation. Despite not achieving the moderate certainty of evidence, it is notable that the four expert consensuses included in this review [13, 20–22] agreed in introducing this test when assessing for clearance following hamstring injuries. Therefore, it can be concluded that assessing pain in soccer-specific drills, especially recreating the mechanism of injury, should be one of the first-priority tests when designing RTP criteria batteries.

Range of motion and psychological readiness criteria have also been implemented in a comparatively larger number of higher-quality studies (e.g., randomized controlled trials and prospective cohort studies) addressing hamstring injuries in soccer players, indicating that these domains have been more consistently described in existing literature, although their independent effect on reinjury risk remains unknown. First, active knee extension [21, 40, 42, 43, 52, 70, 77], passive straight leg raise [13, 20, 21, 42, 43, 52], and Askling-H or active straight leg raise tests [15, 20, 42] are range of motion assessments that could be performed easily with a goniometer at RTP. Nonetheless, these tests, especially the passive straight leg raise test and active knee extension test were elucidated as clinical predictors for reinjury in the baseline assessment of the injured player [167]. Therefore, these tests should be monitored throughout the entire rehabilitation process and could identify patients with increased hamstring reinjury risk [167]. Moreover, those studies performing rehabilitation protocols and presenting similar active knee extension compared with preinjury levels and compared with the contralateral limb showed the lowest range of reinjuries (Table 1). Therefore, this test should be especially considered for inclusion in the assessment when deciding if a player is ready for RTP. Our results also indicate that psychological readiness should play an important role in RTP clearance assessment. Specifically, assessments should evaluate whether the player feels confident to return to sport practice and has no fear of reinjury [13, 20, 21, 23, 41, 43, 46, 78, 115]. A test that can fulfill a triple function (assessment of range of motion, pain during high-speed action, and psychological readiness) could be the Askling-H test, which consists of a rapid hip flexion with full knee extension while the subject is lying supine [15, 50]. Hickey et al. [158] found that the incorporation of the Askling-H test led to the lowest reinjury rates in prospective follow-up in athletes. However, our review presents contrasting findings, as studies published after this systematic review indicate that other specific tests have a lower injury recurrence rate (Table 1). Nonetheless, the Askling-H test remains a useful RTP criteria to assess after hamstring injuries in soccer players on the basis of the existing evidence.

If time and resources allow, assessing additional factors can offer valuable insights for determining a player’s readiness to RTP. Our systematic review documented available evidence regarding RTP, although it is advisable to consider additional factors identified in studies on risk factors for index injury [168–170] and to use practical judgment when designing RTP assessments. This is crucial because significant factors may not be conclusively demonstrated in rigorously designed studies. In any case, future studies should analyze the effects of biomechanical parameters such as adequate lumbopelvic control, anterior pelvic tilt, forward trunk lean, trunk lateral flexion, or maximal hip flexion angle [162], as well as different index injury risk factors in RTP assessment on reinjury rates and mean time to RTP as key variables in rehabilitation.

### Adductors-Related Injury RTP Criteria

For adductors-related injury, the best RCTs implemented absence of pain during resisted contraction (i.e., resisted hip adduction), no pain during on-field or agility tasks, and completing one full team training session as RTP criteria. Pain criteria were also the most cited (68.18% of the existing evidence), followed by strength criteria (36.36% of the existing evidence). Therefore, soccer practitioners should include pain assessments during resisted contraction and during specific on-field tasks and should ensure the completion of one full team training session with high demands before RTP. These tests do not require any resources or technology and are very easy and quick to implement. However, it is important to highlight that pain should be monitored throughout the entire rehabilitation process and not only for RTP [17, 121]. Previous studies indicate that players should be pain-free in resisted isometric outer-range contractions before starting sport-specific training [17, 121]. Further, pain-free status should be maintained during on-field rehabilitation, thus progressively meeting demands of the game [17].

In addition, on the basis of the relatively high number of studies reporting strength criteria, it will be valuable for practitioners to check if the strength levels of the adductors have been restored. It was surprising that all categorized strength-related criteria were not implemented in high-quality RCTs. Nonetheless, it is important to highlight that presenting similar strength levels (i.e., symmetry and similar to preinjury levels) in hip adduction was one of the most cited criterions, included in seven studies [20, 92, 123, 126–128, 133]. Therefore, the implementation of adduction strength levels as one fundamental RTP criteria could be recommended. Serner et al. [17] also highlighted use of the eccentric strength test, especially in late stages of rehabilitation, since it increases consistently as rehabilitation progresses. On the contrary, range of motion outcomes were not useful to determine rehabilitation progress [17]. Therefore, while range of motion and strength-related domains for RTP showed the same certainty of evidence (i.e., very low), it seems fundamental to assess strength levels and especially eccentric adduction strength levels in late stages of rehabilitation. Moreover, similar isokinetic strength was the other strength-related criteria implemented in longitudinal cohort studies [92, 129]. A previous systematic review with meta-analysis showed that decreased isokinetic strength at ~ 119°/s can predict sports-related groin pain [171]. Consequently, we recommend the implementation of hip adduction strength levels in the outer-range position as well as eccentric strength levels and isokinetic hip adduction strength levels for strength RTP criteria on the basis of available evidence. Nonetheless, it is important to note that most of these criteria were reported predominantly in retrospective studies or case series.

According to our results, these tests can be complemented with additional lower-certainty evidence criteria to design a multidimensional assessment. Absence of swelling had a low certainty of evidence for RTP following adductor injuries, with one RCT [47] and one longitudinal cohort study [134] rehabilitating soccer players implementing this criterion. In addition, Jiménez-Rubio et al. [134] specified that this evaluation should be complemented with ultrasound, ensuring the correct alignment of muscle fibers without evidence of edema.

Another important outcome assessed in several studies is soccer-specific ability [17, 20, 87, 121, 122, 133]. Despite not being directly tested in RTP, several studies implemented on-field rehabilitation with several soccer-specific abilities such as kicking, changes of direction, or position-specific drills [47, 61, 134]. Therefore, the qualitative assessment of these abilities plays a significant role in the rehabilitation process. Nonetheless, quantifying performance in key actions such as changes of direction [17, 20, 121, 122, 133] in which the adductor receives a great load during weight acceptance and in the final push-off phase [172, 173] could be crucial to assess RTP readiness. Yet, it is important to address the mechanism of the injury in RTP criteria for a more personalized rehabilitation program. For acute adductor muscle injuries, it is important to distinguish between closed-chain movements such as changing direction or reaching and open-chain movements such as kicking and jumping as the main injury mechanisms in soccer players to design of the rehabilitation programs [173]. Training adductors to increase the capacity to transfer force at lengthening positions as well as training and testing the mechanism in which the injury occurred should constitute a main goal for soccer practitioners [121, 172, 173]. However, it is important to note that our review specifically addresses RTP criteria following acute adductor injuries. Therefore, the implications for RTP decision-making in cases of chronic, long-standing adductor-related groin pain may differ, as the clinical course and rehabilitation demands are not directly comparable.

### Quadriceps Injury RTP Criteria

Pain-related outcomes (i.e., absence of clinical symptoms, no pain during on-field or agility tasks, no pain during and after repeated sprint ability task, and no pain in single-leg jump), completing one full team training, and absence of swelling had the best certainty of evidence for quadriceps injury RTP criteria, although this certainty of evidence was low (i.e., implemented in low quality RCTs and longitudinal cohort studies). This reveals the lack of high-quality evidence reporting quadriceps injury RTP criteria. However, pain criteria were the most cited (76.92% of the existing evidence reporting their assessment), followed by muscle imaging criteria (38.46% of the existing evidence). Soccer practitioners thus should implement low certainty of evidence criteria, complementing evaluation with very low-level evidence domains, while attending to individual player characteristics such as the position or mechanism of injury [174, 175].

In addition, addressing risk factors for quadriceps strains should be a main goal when designing RTP assessment protocols. One previous review [176] highlighted previous injury [177], short height and high body weight [177, 178], limb dominance (i.e., preferred limb for kicking is more susceptible to injury) [5, 177], poor flexibility [178, 179], and strength, especially eccentric knee extension strength and hip flexion strength, [176, 178, 180] as the main intrinsic risk factors for quadriceps injury. This review highlighted flexibility of the quadriceps and strength (i.e., hip flexion and eccentric knee extension) as the main modifiable risk factors for quadriceps strains [176]. Regarding strength assessment, screening of the iliopsoas in terms of detecting weakness or low activation is crucial since the rectus femoris would assume these deficits, increasing risk of injury [176, 180]. Regarding flexibility, appropriate hip extension in addition to knee flexion flexibility is crucial in early swing of the kicking action [176], which is a main mechanism for quadriceps injury in soccer [6, 176, 181]. Another recent systematic review [181] also highlighted previous hamstring injury is an intrinsic nonmodifiable risk factor for quadriceps strain. Therefore, soccer practitioners should emphasize prevention strategies in players with short height and high body weight and those with previous hamstrings and/or quadriceps [176, 181] even after RTP. This should also be addressed throughout the rehabilitation process, since decision-making for RTP clearance in these players should be done even more carefully (e.g., longer time to RTP or addressing more domains in the RTP assessment). Given the highlighted risk factors, soccer practitioners should emphasize assessment of strength levels as well as evaluation of the flexibility of hip flexors and knee extensors as complementary domains identified by our study.

### Calf Injury RTP Criteria

Soccer practitioners should emphasize evaluation of low certainty evidence criteria for RTP since they constitute the best quality of evidence available following calf injury (i.e., implemented in interventional studies such as RCTs or longitudinal cohort studies). However, given the absence of moderate and high certainty of evidence criteria, it is important to highlight that pain criteria and external load progression (77.78% and 55.55% of the existing evidence reporting their use, respectively) were the most cited. Consequently, ensuring no pain during on-field or agility tasks [20, 47, 61], completion of one full team training session [14, 47, 61], and absence of swelling [47] are the most important domains to assess on the basis of the assessment of both qualitative and quantitative available evidence. These tests do not require many resources, especially for pain assessment. However, given the low or very low certainty of evidence of classified domains, future research should establish validation of new RTP criteria through different rehabilitation protocols in RCTs with high methodological quality. When complementing no pain, completion of full team training sessions, and absence of swelling assessments, several criteria with very-low certainty of evidence should be considered. In this line, RTP implemented in longitudinal cohort studies may be prioritized, since they have been in athletes in real-context rehabilitation processes. Therefore, ensuring absence of clinical signs [14, 18, 20, 143], no pain during ambulation [142], and an adequate recovery according to the injury [18, 61, 144] should be prioritized for complementing the aforementioned criteria with low certainty of evidence. Given the lack of high or moderate certainty evidence for RTP criteria following calf injury, expert recommendations should be considered to design future studies. Two expert consensus studies [14, 20] report a wide range of RTP criteria, although both agreed on assessing absence of pain at palpation, similar change of direction performance, similar performance on technical–tactical tasks, and similar calf raise test. These tests should be validated and implemented in interventional studies but could constitute a good starting point, especially given the low resources required for their assessment.

Green et al. [14] highlighted intrinsic and extrinsic risk factors for recurrent calf injuries. One of the most highlighted risk factors and a main intervention during rehabilitation is the ability to perform plyometrics [14, 18, 47]. We were surprised that none of the proposed criteria specifically assessed plyometric ability of the calf, especially for musculotendinous injuries [28] or injuries affecting the tendinous tissue, given the protective role of plyometrics in these injuries [182, 183]. However, tendons mainly act during rapid movements of plantar flexion with the fascicles working isometrically [184, 185]; therefore, the tendon is indirectly assessed in all soccer-specific movements performed at high velocities such as sprinting, jumping, or changing direction. This could help practitioners focus on biomechanics of the injured tissue (i.e., myofascial or musculotendinous), emphasizing the importance of tendon integrity in RTP decisions and incorporating plyometric exercise testing if necessary. Another important highlighted in expert consensus studies is exposure to sport demands in terms of total distance, sprinting, and accelerations and decelerations [14, 20]. Exposing the player to progressive accumulation of these key parameters seems crucial, but practitioners should be careful with high and early volumes of jogging since it is a main mechanism for calf injury, especially if the soleus muscle is affected [186].

### General Lower Limb Muscle Injuries RTP Criteria

All included domains for general lower limb muscle injury RTP criteria had a very low certainty of evidence (i.e., not implemented in RCTs or implemented in longitudinal cohort studies with poor methodological quality). Nonetheless, several domains should be highlighted, since they are applicable to all addressed muscle injuries included in this review and are not addressed in existing literature reporting specific adductors, quadriceps, or calf injury RTP criteria. An interesting outcome to control during rehabilitation is a player’s anthropometry, especially fat percentage [146]. Ensuring < 0.5% change in fat [146] could be an important domain to assess at RTP and during the entire rehabilitation process. This could be of special interest in injuries such as those in the rectus femoris owing to risk factors such as low height and high body mass [176, 181]. In addition, movement quality screening was not reported in any of the studies reporting RTP criteria in specific muscle injuries. Consequently, presenting < 2 cm asymmetry in Y balance test at RTP could be an interesting test for all lower limb muscle injuries [146]. It also is remarkable that two studies [9, 149] reported including assessment of sprinting demands during on-field tasks for RTP to implement following all muscle injuries. While the hamstring is the most affected muscle group in high-intensity running [6, 187], sprinting constitutes one of the most demanding tasks in soccer [87] and thus induces great neuromuscular fatigue, which is a central element for muscle injury [166, 188]. Therefore, practitioners should monitor the volume of sprinting and high-intensity actions during rehabilitation in addition to fatigue levels [174, 175].

### Perspective: Return to Play Is a Process Not an Event

Return to play should be considered a continuum rather than a single event, as emphasized in the 2016 Bern consensus statement [189]. The return to sport process should be understood as a continuum comprising three distinct but interrelated phases: return to participation (resuming modified or partial training), return to sport (resuming competition, though not necessarily at preinjury performance level), and return to performance (achieving or surpassing preinjury performance). Within this continuum, progression criteria should be adapted to each stage, for example, pain and range of motion in early rehabilitation, strength and functional capacity in intermediate stages, and sport-specific demands and training exposure prior to full return. Importantly, given that players often present residual deficits even after returning to competition [190], rehabilitation should continue beyond this point [191] to ensure progression toward the return to performance stage described in the consensus statement.

### Limitations

It is important to highlight the limitations of this systematic review and evidence assessment. First, the limited number of women included in the reviewed studies makes it challenging to generalize our results to female soccer players. Therefore, future studies should address differences in RTP assessment between male and female soccer players, considering biomechanical and neuromuscular differences between men and women [192, 193]. Another significant limitation is the very low certainty of evidence demonstrated by most included domains, thus making it challenging to establish definitive conclusions about RTP criteria. Importantly, the GRADE certainty ratings used in this review characterize the robustness of the evidence base describing each RTP domain, but they do not demonstrate that any specific RTP criterion is effective for reducing reinjury risk or time to RTP. Future research with well-designed RCTs with prospective follow-up is needed, using various criteria to determine RTP clearance. In addition, it would be interesting to analyze differences in reinjury risk and time to RTP using different criteria with a similar rehabilitation program for muscle injuries, since effectiveness of the analyzed criteria in this study is highly influenced by the previously designed rehabilitation protocol; thus, isolating the effects of RTP criteria is difficult based on available evidence. Therefore, reinjury rates and mean time to RTP ranges for each criterion presented in this study should be interpreted with caution, since they do not reflect the direct effect of considering RTP criteria. Another limitation of this review is the deviation from the original PROSPERO protocol, which initially specified the inclusion of interventional studies only. To provide a more comprehensive overview of RTP criteria, we also collected evidence from cross-sectional studies and expert consensus. Nevertheless, only interventional evidence is presented in the primary focus of this manuscript, with noninterventional evidence reported in the supplementary materials for completeness. Finally, defined domains were created based on the authors’ perspectives and expertise, potentially affecting assessed levels of evidence. Nevertheless, our classifications align with those in two previous systematic reviews of RTP criteria on hamstring injuries [12, 158], one recent scoping review [194], as well as with expert consensus classifications [14, 20, 133].

## Conclusions

This systematic review of muscle injuries in soccer players encourages practitioners to select the most suitable RTP assessment protocol. Strength and pain criteria are the most cited in literature for hamstring injuries clearance, specifically presenting similar limb to limb knee flexors/extensors strength levels when compared with preinjury and the contralateral limb, as well as presenting no pain during soccer-specific drills. Tests evaluating range of motion (i.e., active knee extension, passive and active straight leg raise, and Askling-H tests) along with subjective readiness assessments were implemented in the RCTs with the highest methodological quality for guiding RTP decisions following hamstring injuries in soccer players. For adductors-related injury, pain assessments and completing at least one full team training session were implemented in RCTS with the highest methodological quality, while correct muscle imaging assessment, especially with absence of swelling, was implemented in longitudinal cohort studies or RCTs with poor methodological quality. It is important to check strength levels following adductors injury given the high number of studies reporting their assessment. For quadriceps injury, pain assessments (the most cited criteria in literature), completing at least one full team training session, and correct muscle imaging assessment, especially with absence of swelling, were implemented in RCTs but with poor methodological quality. For calf injury, pain assessments and correct muscle imaging assessment, especially with absence of swelling, were the criteria implemented in RCTs with poor methodological quality. Pain-related criteria were the most frequently reported in the literature.

## Supplementary Information

Below is the link to the electronic supplementary material.

Supplementary file1 (DOCX 15 KB)

Supplementary file2 (XLSX 160 KB)

Supplementary file3 (DOCX 247 KB)

Supplementary file4 (DOCX 167 KB)

## Declarations

### Data Sharing

All data relevant to the study are included in the article or are available as supplementary files.
