# Supplementary material for: Return-to-Play Criteria Following Lower Limb Muscle Injuries in Soccer: A Systematic Review with Evidence Synthesis
Source: Sports Med. 2026 Mar 18;56(6):1433–65. doi: 10.1007/s40279-026-02404-9 (PMC13260053; doi:10.1007/s40279-026-02404-9)
Supplement: Supplementary file 1 — Supplementary file1 (DOCX 15 KB) [file 40279_2026_2404_MOESM1_ESM.docx]

**Title:** Return-to-play criteria following lower-limbs muscle injuries in soccer. A systematic review with evidence synthesis

**Journal name:** Sports Medicine

**Author names & affiliations:**

Javier Pecci^1^*, Nicol van Dyk^2,3^, Gregory D. Myer^4,5,6,7,8^, Borja Sañudo^1^

^1^ Department of Physical Education and Sport, University of Seville, Seville, Spain

^2^ Section Sports Medicine, Faculty of Health Sciences, University of Pretoria, Pretoria, South Africa

^3^ School of Public Health, Physiotherapy and Sport Sciences, University College Dublin, Dublin, Ireland

^4^ Sports Performance And Research Center (SPARC), Emory University School of Medicine, Flowery Branch, GA, USA

^5^ Department of Orthopaedics, Emory University School of Medicine, Atlanta, GA, USA

^6^ Wallace H. Coulter Department of Biomedical Engineering, Georgia Institute of Technology & Emory University, Atlanta, GA, USA

^7^ The Micheli Center for Sports Injury Prevention, Waltham, MA, USA

^8^ Youth Physical Development Centre, Cardiff Metropolitan University, Wales, UK

**E-mail address (corresponding author):** [jpecci@us.es](mailto:jpecci@us.es)

Online Supplementary File 1

Specific search strategies

**PubMed:** Search: ((soccer OR football OR sport* OR player*) AND (rehab* OR treat* OR reconditioning OR "return to play" OR "return to sport" OR "return-to-play" OR "return-to-sport" OR recover* OR intervention* OR progress* OR therap* OR manag*) AND ("soft tissue" OR "soft-tissue" OR muscle* OR muscular OR tendon OR hamstring* OR "biceps femoris" OR semitendinosus OR semimembranosus OR quadriceps OR "rectus femoris" OR "vastus lateralis" OR "vastus intermedius" OR "vastus medialis" OR calf OR "triceps surae" OR soleus OR gastrocnemius OR adductor* OR groin) AND (tear OR injur* OR avulsion OR strain OR rupture OR trauma* OR pain))

Published date: Database start–20240310

**Web of Science:** TOPIC: ((soccer OR football OR sport* OR player*) AND (rehab* OR treat* OR reconditioning OR "return to play" OR "return to sport" OR "return-to-play" OR "return-to-sport" OR recover* OR intervention* OR progress* OR therap* OR manag*) AND ("soft tissue" OR "soft-tissue" OR muscle* OR muscular OR tendon OR hamstring* OR "biceps femoris" OR semitendinosus OR semimembranosus OR quadriceps OR "rectus femoris" OR "vastus lateralis" OR "vastus intermedius" OR "vastus medialis" OR calf OR "triceps surae" OR soleus OR gastrocnemius OR adductor* OR groin) AND (tear OR injur* OR avulsion OR strain OR rupture OR trauma* OR pain))

Published date: Database start–20240310

**SPORTDiscus:** TX (soccer OR football OR sport* OR player*) AND TX (rehab* OR treat* OR reconditioning OR "return to play" OR "return to sport" OR "return-to-play" OR "return-to-sport" OR recover* OR intervention* OR progress* OR therap* OR manag*) AND TX ("soft tissue" OR "soft-tissue" OR muscle* OR muscular OR tendon OR hamstring* OR "biceps femoris" OR semitendinosus OR semimembranosus OR quadriceps OR "rectus femoris" OR "vastus lateralis" OR "vastus intermedius" OR "vastus medialis" OR calf OR "triceps surae" OR soleus OR gastrocnemius OR adductor* OR groin) AND TX (tear OR injur* OR avulsion OR strain OR rupture OR trauma* OR pain)

Published date: Database start–20240310

**CINAHL: (**(soccer OR football OR sport* OR player*) AND (rehab* OR treat* OR reconditioning OR "return to play" OR "return to sport" OR "return-to-play" OR "return-to-sport" OR recover* OR intervention* OR progress* OR therap* OR manag*) AND ("soft tissue" OR "soft-tissue" OR muscle* OR muscular OR tendon OR hamstring* OR "biceps femoris" OR semitendinosus OR semimembranosus OR quadriceps OR "rectus femoris" OR "vastus lateralis" OR "vastus intermedius" OR "vastus medialis" OR calf OR "triceps surae" OR soleus OR gastrocnemius OR adductor* OR groin) AND (tear OR injur* OR avulsion OR strain OR rupture OR trauma* OR pain))

Published date: Database start–20240310

**EMBASE:** ((soccer OR football OR sport* OR player*) AND (rehab* OR treat* OR reconditioning OR "return to play" OR "return to sport" OR "return-to-play" OR "return-to-sport" OR recover* OR intervention* OR progress* OR therap* OR manag*) AND ("soft tissue" OR "soft-tissue" OR muscle* OR muscular OR tendon OR hamstring* OR "biceps femoris" OR semitendinosus OR semimembranosus OR quadriceps OR "rectus femoris" OR "vastus lateralis" OR "vastus intermedius" OR "vastus medialis" OR calf OR "triceps surae" OR soleus OR gastrocnemius OR adductor* OR groin) AND (tear OR injur* OR avulsion OR strain OR rupture OR trauma* OR pain))

Published date: Database start–20240310

**PsycInfo:** ((soccer OR football OR sport* OR player*) AND (rehab* OR treat* OR reconditioning OR "return to play" OR "return to sport" OR "return-to-play" OR "return-to-sport" OR recover* OR intervention* OR progress* OR therap* OR manag*) AND ("soft tissue" OR "soft-tissue" OR muscle* OR muscular OR tendon OR hamstring* OR "biceps femoris" OR semitendinosus OR semimembranosus OR quadriceps OR "rectus femoris" OR "vastus lateralis" OR "vastus intermedius" OR "vastus medialis" OR calf OR "triceps surae" OR soleus OR gastrocnemius OR adductor* OR groin) AND (tear OR injur* OR avulsion OR strain OR rupture OR trauma* OR pain))

Published date: Database start–20240310
