# Supplementary material for: Return-to-Play Criteria Following Lower Limb Muscle Injuries in Soccer: A Systematic Review with Evidence Synthesis
Source: Sports Med. 2026 Mar 18;56(6):1433–65. doi: 10.1007/s40279-026-02404-9 (PMC13260053; doi:10.1007/s40279-026-02404-9)
Supplement: Supplementary file 3 — Supplementary file3 (DOCX 247 KB) [file 40279_2026_2404_MOESM3_ESM.docx]

**Title:** Return-to-play criteria following lower-limbs muscle injuries in soccer. A systematic review with evidence synthesis

**Journal name:** Sports Medicine

**Author names & affiliations:**

Javier Pecci^1^*, Nicol van Dyk^2,3^, Gregory D. Myer^4,5,6,7,8^, Borja Sañudo^1^

^1^ Department of Physical Education and Sport, University of Seville, Seville, Spain

^2^ Section Sports Medicine, Faculty of Health Sciences, University of Pretoria, Pretoria, South Africa

^3^ School of Public Health, Physiotherapy and Sport Sciences, University College Dublin, Dublin, Ireland

^4^ Sports Performance And Research Center (SPARC), Emory University School of Medicine, Flowery Branch, GA, USA

^5^ Department of Orthopaedics, Emory University School of Medicine, Atlanta, GA, USA

^6^ Wallace H. Coulter Department of Biomedical Engineering, Georgia Institute of Technology & Emory University, Atlanta, GA, USA

^7^ The Micheli Center for Sports Injury Prevention, Waltham, MA, USA

^8^ Youth Physical Development Centre, Cardiff Metropolitan University, Wales, UK

**E-mail address (corresponding author):** [jpecci@us.es](mailto:jpecci@us.es)

Online Supplementary File 3

Evidence on RTP criteria for non-interventional studies.

**Table S3.1.** Summary of results of the included studies filtered by return-to-play criteria and type (i.e., design) of study following hamstring injuries for non-interventional studies. Sentences accompanied by numbers in brackets represent summary of the evidence for a specific criterion/type of study and the studies reporting it, respectively. GPS: global positioning system; MRI: magnetic resonance imaging. The term “similar” here refers to similar to pre-injury levels and similar to the contralateral limb.

| **RTP criteria** | **Cross-sectional** | **Expert consensus** |
| --- | --- | --- |
| Pain | No pain [78–83,114,117,118] | No pain [13,20–22] |
|  |  |  |
|  |  |  |
| External load progression | Meet competition demands [84,85,117] | Recover GPS training profile [13,20–22,119] |
| Jumping kinetics and kinematics | Similar CMJ, triple hop and jumping kinetics [86–88,111] |  |
| Strength | Similar strength and fatigability [78,81,82,86,89–99,110,113,117,118] | Similar knee flexors / extensors strength [13,20–22] |
| Range of motion | <10% asymmetry in hamstrings flexibility [78,92,114] | Similar range of motion [13,20,21] |
| Biomechanics | Symmetry in kicking and sprinting biomechanics [100,101] | Adequate lumbopelvic control [21] |
| Muscle activation | Similar and good hamstrings, gluteal and trunk muscles activation [87,96,102–104,111,113] |  |
| Muscle imaging | Maturity of the scar and similar muscle architecture [90,93,95,105,106,112] | Hiperintensity reduction of at least 70% [20] |
| Rate of Perceived Exertion | Lower Rate of Perceived Exertion after RSA [88] |  |
| Psychology | Similar perceived hamstrings function [79,114] | Subjective readiness for completion and no fear of re-injury [13,20–22] |
| Healing | Respect biological healing of the tissue [106] |  |
| Agility / Soccer-specific ability | Recover maximal horizontal force and fatigability [107,108] | Similar sprinting, braking and Illinois test [13,21] |
| Muscle tissue contractile and sensorial properties | Similar stiffness and proprioceptive capacity [83,87,109] |  |
| Fitness |  | Similar metabolic performance [21] |

**Table S3.2** Summary of results of included studies filtered by return-to-play criteria, domain, and type (i.e., design) of study following adductors injury for non-interventional studies. RTP: return-to-play; GPS: global positioning system; HAGOS: Copenhagen Hip and Groin Outcome Score; CMJ: countermovement jump; RSA: Repeated Sprint Ability; LEFS: Lower Extremity Functional Scale.

| **RTP criteria** | **Cross-sectional** | **Expert consensus** |
| --- | --- | --- |
| Pain criteria |  | Absence of pain [12,45] |
| Biomechanics criteria | Similar pre-injury and contralateral anterior and total pelvic tilt [46,47] | Good movement quality [45] |
| Football-specific abilities | Low pre-post difference in Squat Jump and CMJ after RSA with lower pre-injury RPE [20] | Similar field performance than pre-injury [12,45] |
| External load progression |  | <10% of GPS performance in training compared to pre-injury levels [12] |
| Strength | Asymmetry of <10% between injured and uninjured limbs in hip adduction strength [48] | Similar isometric and eccentric adductors strength [12] |
| Muscle imaging criteria |  | Hyperintensity reduction of 70% and check low signal intensity [12] |
| Subjective outcomes | Improvement in LEFS and Global Rating of Charge [49] | No fear of re-injury and improvement in HAGOS scale [12,45] |
| Range of motion criteria |  | Similar hip abduction range of motion [12] |

**Table S3.3** Summary of results of included studies filtered by return-to-play criteria, domain, and type (i.e., design) of study following quadriceps injury for non-interventional studies. RTP: return-to-play; RCT: randomized controlled trial; GPS: global positioning system; MRI: magnetic resonance imaging; HSR: High-speed running; EMG: electromyography.

| **RTP criteria** | **Cross-sectional** | **Expert consensus** |
| --- | --- | --- |
| Pain criteria |  | Absence of pain at palpation, stretching and resisted contraction [20] |
| Football-specific abilities |  | Similar pre-injury braking, Illinois Agility and Kicking test [20] |
| External load progression |  | <10% of GPS performance in training compared to pre-injury levels [20] |
| Strength |  | Recover pre-injury and contralateral knee extensor strength [20] |
| Muscle imaging criteria |  | Hyperintensity reduction of 70% and check low signal intensity [20] |
| Subjective outcomes |  | Low levels of anxiety, apprehension, fear of failure and/or fear of reinjury [20] |
| Range of motion criteria |  | Similar hip abduction range of motion [20] |
| Muscle activation criteria | Similar proximal rectus femoris EMG activation with special focus in hip flexion [137,142] |  |

**Table S3.4** Summary of results of included studies filtered by return-to-play criteria, domain, and type (i.e., design) of study following calf injury for non-interventional studies. RTP: return-to-play; RCT: randomized controlled trial; GPS: global positioning system; MRI: magnetic resonance imaging; CoD: change of direction.

| **RTP criteria** | **Cross-sectional** | **Expert consensus** |
| --- | --- | --- |
| Pain criteria |  | Absence of pain [14,20] |
| Football-specific abilities |  | Similar on-field performance with emphasis on CoD and jumping performance [14,20] |
| External load progression |  | Similar pre-injury full team training GPS external load profile [14,20] |
| Strength |  | Similar strength profile of the player [14,20] |
| Muscle imaging criteria | Large scar thickness in ultrasound and similar Shear Wave Elastography [146] | Hyperintensity reduction of 70% and check low signal intensity [14] |
| Subjective outcomes |  | Low levels of anxiety, apprehension, fear of failure and/or fear of reinjury [20] |
| Range of motion criteria |  | Similar ankle flexibility test [20] |
| Healing criteria | Check the Return to Play in a normal interval for the grade of the injury [145] |  |

**Table S3.5** Summary of results of included studies filtered by return-to-play criteria, domain, and type (i.e., design) of study following general lower limb muscle injury for non-interventional studies. RTP: return-to-play; RCT: randomized controlled trial; GPS: global positioning system; MRI: magnetic resonance imaging.

| **RTP criteria** | **Cross-sectional** | **Expert consensus** |
| --- | --- | --- |
| Pain criteria |  | Pain-free stretching, contractions and basic sports techniques [155] |
| Football-specific abilities | Recover maximal speed and match kicking load [9,149] |  |
| External load progression | Recover maximal speed demands [9] |  |
| Jumping performance | Low asymmetry in concentric and eccentric impulse, peak force and force at zero velocity [154] |  |
| Muscle imaging | No connective tissue gap, loss of tendon tension or intramuscular oedema [152] |  |
| Psychology |  |  |
| Range of motion |  | Similar range of motion compared to the unaffected limb [155] |
