# Supplementary material for: Return-to-Play Criteria Following Lower Limb Muscle Injuries in Soccer: A Systematic Review with Evidence Synthesis
Source: Sports Med. 2026 Mar 18;56(6):1433–65. doi: 10.1007/s40279-026-02404-9 (PMC13260053; doi:10.1007/s40279-026-02404-9)
Supplement: Supplementary file 4 — Supplementary file4 (DOCX 167 KB) [file 40279_2026_2404_MOESM4_ESM.docx]

**Title:** Return-to-play criteria following lower-limbs muscle injuries in soccer. A systematic review with evidence synthesis

**Journal name:** Sports Medicine

**Author names & affiliations:**

Javier Pecci^1^*, Nicol van Dyk^2,3^, Gregory D. Myer^4,5,6,7,8^, Borja Sañudo^1^

^1^ Department of Physical Education and Sport, University of Seville, Seville, Spain

^2^ Section Sports Medicine, Faculty of Health Sciences, University of Pretoria, Pretoria, South Africa

^3^ School of Public Health, Physiotherapy and Sport Sciences, University College Dublin, Dublin, Ireland

^4^ Sports Performance And Research Center (SPARC), Emory University School of Medicine, Flowery Branch, GA, USA

^5^ Department of Orthopaedics, Emory University School of Medicine, Atlanta, GA, USA

^6^ Wallace H. Coulter Department of Biomedical Engineering, Georgia Institute of Technology & Emory University, Atlanta, GA, USA

^7^ The Micheli Center for Sports Injury Prevention, Waltham, MA, USA

^8^ Youth Physical Development Centre, Cardiff Metropolitan University, Wales, UK

**E-mail address (corresponding author):** [jpecci@us.es](mailto:jpecci@us.es)

**HAMSTRING INJURIES RTP CRITERIA**

**Table S3.1 Summary of risk of bias and level of evidence assessment for return to play criteria implemented in randomized controlled trials and thus starting at high level of evidence**

| RTP criteria | Domain | Study | Risk of bias assessment | | | | | | | GRADE level of evidence | | | | | | |
| --- | --- | --- | --- | --- | --- | --- | --- | --- | --- | --- | --- | --- | --- | --- | --- | --- |
|  |  |  | 1 | 2 | 3 | 4 | 5 | Overall | Risk of bias | | Inconsistency | Indirectness | Imprecise evidence | Publication bias | Level of evidence |  |
| No pain | No pain at palpation | A Hamid et al. (2014) | 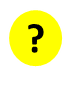 | 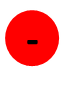 | 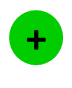 | 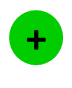 | 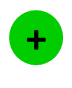 | 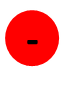 | 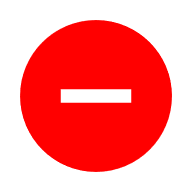 | | 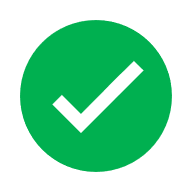 | 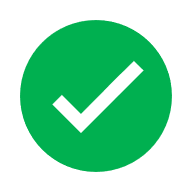 | 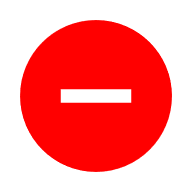 | 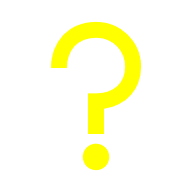 | Low |  |
|  |  | Mendiguchia et al. (2017) | 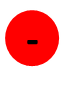 | 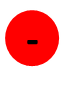 | 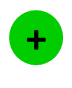 | 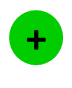 | 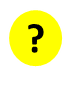 | 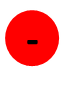 |  |  |  |  |  |  |  |  |
|  |  | Slider et al. (2013) | 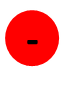 | 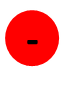 | 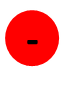 | 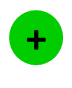 | 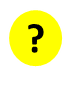 | 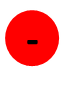 |  |  |  |  |  |  |  |  |
|  |  | Medeiros et al. (2020) | 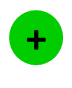 | 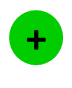 | 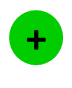 | 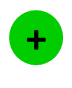 | 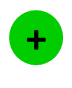 | 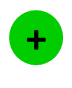 |  |  |  |  |  |  |  |  |
|  |  | Hickey et al. (2020) | 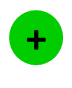 | 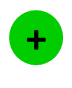 | 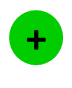 | 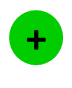 | 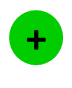 | 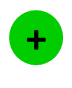 |  |  |  |  |  |  |  |  |
|  | No pain during football-specific actions | Sherry and Best (2004) | 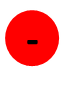 | 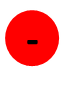 | 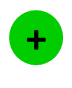 | 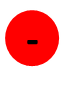 | 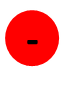 | 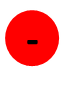 | 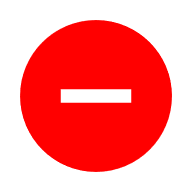 | | 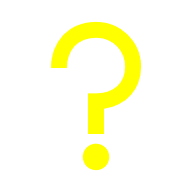 | 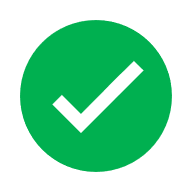 | 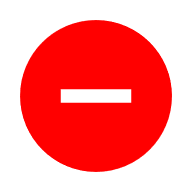 | 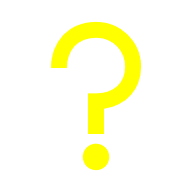 | Low |  |
|  |  | Medeiros et al. (2020) | 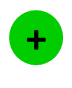 | 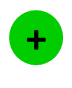 | 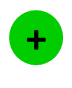 | 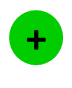 | 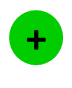 | 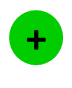 |  |  |  |  |  |  |  |  |
|  |  | Hickey et al. (2020) | 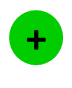 | 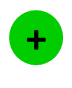 | 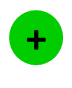 | 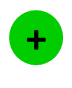 | 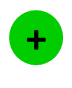 | 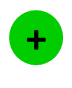 |  |  |  |  |  |  |  |  |
|  |  | Bayer et al. (2018) | 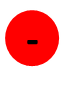 | 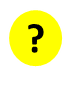 | 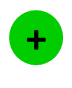 | 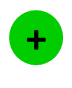 | 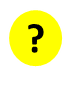 | 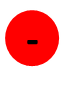 |  |  |  |  |  |  |  |  |
|  |  | Vermeulen et al. (2022) | 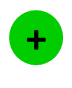 | 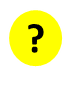 | 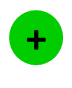 | 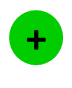 | 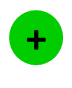 | 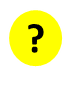 |  |  |  |  |  |  |  |  |
|  |  | Hägglund et al. (2007) | 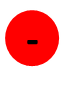 | 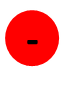 | 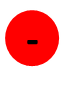 | 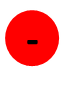 | 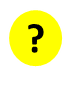 | 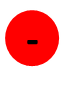 |  |  |  |  |  |  |  |  |
|  |  | Hamilton et al. (2015) | 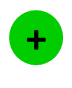 | 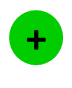 | 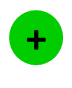 | 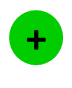 | 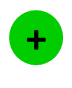 | 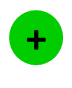 |  |  |  |  |  |  |  |  |
|  |  | Bezuglov et al. (2019) | 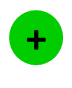 | 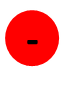 | 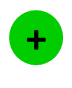 | 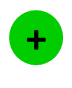 | 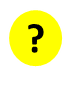 | 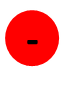 |  |  |  |  |  |  |  |  |
|  |  | Mendiguchia et al. (2017) | 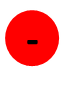 | 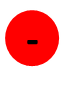 | 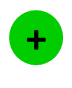 | 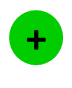 | 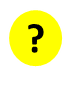 | 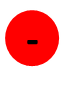 |  |  |  |  |  |  |  |  |
|  |  | González-Iglesias et al. (2023) | 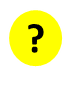 | 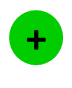 | 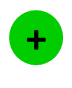 | 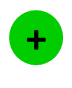 | 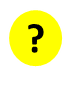 | 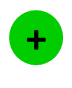 |  |  |  |  |  |  |  |  |
|  | No pain during strengthening or stretching | Mendiguchia et al. (2017) |  |  |  |  |  |  |  | |  |  |  |  | Low |  |
|  |  | Askling et al. (2014) |  |  |  |  |  |  |  |  |  |  |  |  |  |  |
|  |  | Medeiros et al. (2020) |  |  |  |  |  |  |  |  |  |  |  |  |  |  |
|  |  | Vermeulen et al. (2022) |  |  |  |  |  |  |  |  |  |  |  |  |  |  |
|  |  | Hickey et al. (2020) |  |  |  |  |  |  |  |  |  |  |  |  |  |  |
|  |  | A Hamid et al. (2014) |  |  |  |  |  |  |  |  |  |  |  |  |  |  |
| Jumping kinetics and kinematics | Similar jumping performance | Sherry and Best (2004) |  |  |  |  |  |  |  | |  |  |  |  | Low |  |
|  |  | Medeiros et al. (2020) |  |  |  |  |  |  |  |  |  |  |  |  |  |  |
|  |  | Mendiguchia et al. (2017) |  |  |  |  |  |  |  |  |  |  |  |  |  |  |
|  |  | González-Iglesias et al. (2023) |  |  |  |  |  |  |  |  |  |  |  |  |  |  |
| Strength | Similar knee flexor/extensor strength | Askling et al. (2013) |  |  |  |  |  |  |  | |  |  |  |  | Low |  |
|  |  | Slider et al. (2013) |  |  |  |  |  |  |  |  |  |  |  |  |  |  |
|  |  | Mendiguchia et al. (2017) |  |  |  |  |  |  |  |  |  |  |  |  |  |  |
|  |  | Hamilton et al. (2015) |  |  |  |  |  |  |  |  |  |  |  |  |  |  |
|  |  | Vermeulen et al. (2022) |  |  |  |  |  |  |  |  |  |  |  |  |  |  |
|  |  | A Hamid et al. (2014) |  |  |  |  |  |  |  |  |  |  |  |  |  |  |
|  |  | González-Iglesias et al. (2023) |  |  |  |  |  |  |  |  |  |  |  |  |  |  |
| Range of motion | Similar active knee extension test | A Hamid et al. (2014) |  |  |  |  |  |  |  | |  |  |  |  | Moderate |  |
|  |  | Medeiros et al. (2020) |  |  |  |  |  |  |  |  |  |  |  |  |  |  |
|  |  | Hickey et al. (2020) |  |  |  |  |  |  |  |  |  |  |  |  |  |  |
|  | Similar passive straight leg raise test | Medeiros et al. (2020) |  |  |  |  |  |  |  | |  |  |  |  | Moderate |  |
|  |  | Hickey et al. (2020) |  |  |  |  |  |  |  |  |  |  |  |  |  |  |
|  |  | González-Iglesias et al. (2023) |  |  |  |  |  |  |  | |  |  |  |  |  |  |
|  | Similar performance in Askling-H or ASLR test | Medeiros et al. (2020) |  |  |  |  |  |  |  | |  |  |  |  | Moderate |  |
|  |  | Mendiguchia et al. (2017) |  |  |  |  |  |  |  |  |  |  |  |  |  |  |
| Psychology | Subjective readiness | Hickey et al. (2020) |  |  |  |  |  |  |  | |  |  |  |  | Moderate |  |
|  |  | Slider et al. (2013) |  |  |  |  |  |  |  |  |  |  |  |  |  |  |
|  |  | Vermeulen et al. (2022) |  |  |  |  |  |  |  |  |  |  |  |  |  |  |
| Muscle imaging | Maturity of the scar tissue in MRI or ultrasound | Hägglund et al. (2007) |  |  |  |  |  |  |  | |  |  |  |  | Low |  |
|  |  | González-Iglesias et al. (2023) |  |  |  |  |  |  |  |  |  |  |  |  |  |  |

**Table S3.2 Summary of risk of bias and level of evidence for return to play criteria implemented in longitudinal cohort studies and thus starting at high level of evidence**

| RTP criteria | Domain | Study | SIGN Checklist | | | | | | | | | | | | | | | GRADE level of evidence | | | | | | |
| --- | --- | --- | --- | --- | --- | --- | --- | --- | --- | --- | --- | --- | --- | --- | --- | --- | --- | --- | --- | --- | --- | --- | --- | --- |
|  |  |  | 1 | 2 | 3 | 4 | 5 | 6 | 7 | 8 | 9 | 10 | 11 | 12 | 13 | 14 | Risk of bias | | Inconsistency | Indirectness | Imprecise evidence | Publication bias | Level of evidence |  |
| External load progression | Progression and control of match exposure | Jiménez-Rubio et al. (2020) |  | N/A |  | N/A |  | N/A |  |  |  |  |  | N/A |  |  |  | |  |  |  |  | Very low |  |
|  |  | Whiteley et al. (2021) |  | N/A |  | N/A |  | N/A |  | N/A |  |  |  | N/A |  |  |  |  |  |  |  |  |  |  |
|  | At least 1-5 full training sessions | Hickey et al. (2022) |  | N/A |  | N/A |  | N/A |  |  |  |  |  |  |  |  |  | |  |  |  |  | Very low |  |
|  |  | Reurink et al. (2014) |  | N/A |  | N/A |  | N/A |  |  |  |  |  | N/A |  |  |  |  |  |  |  |  |  |  |
|  |  | Reurink et al. (2015) |  | N/A |  | N/A |  | N/A |  |  |  |  |  | N/A |  |  |  |  |  |  |  |  |  |  |
|  | Similar GPS profile in training sessions | Jiménez-Rubio et al. (2018) |  | N/A |  | N/A |  | N/A |  |  |  |  |  | N/A |  |  |  | |  |  |  |  | Very low |  |
|  |  | Pruna et al. (2019) |  | N/A |  | N/A |  | N/A |  |  |  |  |  | N/A |  |  |  |  |  |  |  |  |  |  |
| Range of motion | Similar unspecified range of motion | Schmitt et al. (2020) |  | N/A |  | N/A |  | N/A |  |  |  |  |  | N/A |  |  |  | |  |  |  |  | Very low |  |
|  |  | Van der Made et al. (2017) |  | N/A |  | N/A |  | N/A |  |  |  |  |  | N/A |  |  |  |  |  |  |  |  |  |  |

Item fulfilled

Some concerns or unclear

Item not fulfilled

Fulfillment: Level of evidence not downgraded for this outcome

Unclear fulfillment: Level of evidence not downgraded for this outcome

No fulfillment: Level of evidence downgraded by one level for this outcome

No fulfillment: Level of evidence downgraded by two levels for this outcome

Level of evidence was rated as high, moderate, low or very low [1]. Level of evidence started at high when RTP criteria were based on randomized controlled trials (RCTs i.e., 2 or more studies) or low if best evidence was based on longitudinal cohort studies [1]. Since RTP criteria is an outcome within the broader rehabilitation process, and no effect estimates exist for key outcomes (i.e., time to return to play and re-injuries), the level of evidence was assessed based on current recommendations for outcomes without effect sizes and using a narrative synthesis of the results [2]. An example of GRADE application for each domain based on existing evidence [2] can be found on Table S3.3. Level of evidence was downgraded if :1) most studies (i.e., >50%) that reported RTP criteria presented high risk of bias (downgraded by one level) or all RCTs presented high risk of bias (downgraded by two levels) according to the Grade handbook [1]; 2) there was inconsistency in specific RTP criteria across different domains (see Supplementary File 2); or 3) imprecision was found. Evidence was downgraded by one level if there were <800 participants in a comparison [3] and by two levels if there also was no clear direction of effects. All studies were pre-judged at low risk of indirectness, since the eligibility criteria used guaranteed relevant populations and interventions, and there was no need to use surrogate outcomes. Risk of publication bias could not be explicitly assessed due to the reduced number of studies (i.e., <10) available for each domain, but authors judged the publication bias in a narrative way following previous recommendations for time to return to play and re-injuries results [2]. When evidence was based on RCTs, the Risk of Bias 2 tool [4] was used; when evidence was based on longitudinal cohort studies, the Scottish Intercollegiate Guidelines Network checklist for cohort studies [5] was used. When evidence was based on cross-sectional, expert consensus, or case studies, the level of evidence was rated as very low.

**Table S3.3 An example of the application of modified GRADE for rating certainty of evidence of the studies reporting each RTP criterion based on previous recommendations [2].**

| Outcome: No pain at palpation | | |
| --- | --- | --- |
| GRADE domain | Judgement | Concerns about certainty of domains |
| Methodological limitations of the studies | Three out of five studies reporting this criterion for RTP presented high overall risk of bias, with special concerns on deviations from the intended interventions. Several concerns on blinded evaluators and participants were found, with one study [6] presenting major bias for the randomization process, due to deviations from the intended interventions and bias due to missing outcome data. | Serious |
| Inconsistency | Pain at palpation was assessed in a very similar way in all included studies reporting this criterion for RTP. The assessment of re-injuries and time to return to play was similar in all included studies for this criterion. | Not serious |
| Indirectness | Given our eligibility criteria and the created domains for assessment based on existing evidence, indirectness should be rated with no concerns. | Not serious |
| Imprecision | Studies reporting this criterion included less than 800 soccer players. | Serious |
| Publication bias | Publication bias was not suspected, since wide ranges of re-injuries were reported for included studies (0% to 23.07%) and mean time to return to play (15 to 28.8 days). | Not suspected |

**ADDUCTOR, QUADRICEPS, CALF INJURIES AND GENERAL RTP CRITERIA**

**Table S3.4 Risk of bias assessment for randomized controlled trials based on Risk of Bias 2 scale**

| Study | Risk of Bias 2 assessment | | | | | | |
| --- | --- | --- | --- | --- | --- | --- | --- |
|  | 1 | 2 | 3 | 4 | 5 | Overall |  |
| Hölmich et al. (1999) |  |  |  |  |  |  |  |
| Häglund et al. (2017) |  |  |  |  |  |  |  |
| Bayer et al. (2018) |  |  |  |  |  |  |  |

**Table S3.5 Risk of bias assessment for longitudinal cohort studies based on Scottish Intercollegiate Guidelines Network (SIGN) Checklist 3**

| Study | SIGN Checklist | | | | | | | | | | | | | | |
| --- | --- | --- | --- | --- | --- | --- | --- | --- | --- | --- | --- | --- | --- | --- | --- |
|  | 1 | 2 | 3 | 4 | 5 | 6 | 7 | 8 | 9 | 10 | 11 | 12 | 13 | 14 |  |
| Mechó et al. (2023) |  | N/A |  | N/A |  | N/A |  |  |  |  |  | N/A |  |  |  |
| Serner et al. (2020a) |  | N/A |  | N/A |  | N/A |  |  |  |  |  | N/A |  |  |  |
| Serner et al. (2021) |  | N/A |  | N/A |  | N/A |  |  |  |  |  |  |  |  |  |
| Serner et al. (2020b) |  | N/A |  | N/A |  | N/A |  |  |  |  |  | N/A |  |  |  |
| Nagamoto (2020) |  | N/A |  | N/A |  | N/A |  |  |  |  |  | N/A |  |  |  |
| Tak et al. (2018) |  | N/A |  | N/A |  | N/A |  |  |  |  |  | N/A |  |  |  |
| Jiménez-Rubio et al. (2021) |  | N/A |  | N/A |  | N/A |  |  |  |  |  | N/A |  |  |  |
| Corazza et al. (2013) |  | N/A |  | N/A |  | N/A |  |  |  |  |  | N/A |  |  |  |
| Pezzota et al. (2018) |  | N/A |  | N/A |  | N/A |  |  |  |  |  | N/A |  |  |  |
| Pedret et al. (2015) |  | N/A |  | N/A |  | N/A |  |  |  |  |  | N/A |  |  |  |
| Kwak et al. (2006) |  | N/A |  | N/A |  | N/A |  |  |  |  |  | N/A |  |  |  |
| Balius et al. (2009) |  | N/A |  | N/A |  | N/A |  |  |  |  |  | N/A |  |  |  |
| Shimozaki et al. (2022) |  | N/A |  | N/A |  | N/A |  |  |  |  |  | N/A |  |  |  |
| Valera-Garrido et al. (2020) |  | N/A |  | N/A |  | N/A |  |  |  |  |  |  |  |  |  |
| Raya-González et al. (2019) |  | N/A |  | N/A |  | N/A |  |  |  |  |  |  |  |  |  |
| Gómez-Piqueras et al. (2018) |  | N/A |  | N/A |  | N/A |  |  |  |  |  |  |  |  |  |
| Bezuglov et al. (2022) |  | N/A |  | N/A |  | N/A |  |  |  |  |  |  |  |  |  |
| Portillo et al. (2020) |  | N/A |  | N/A |  | N/A |  | N/A | N/A |  |  |  |  |  |  |
| Fuller et al. (2006) |  | N/A |  | N/A |  | N/A |  |  |  |  |  | N/A |  |  |  |
| Bengtsson et al. (2019) |  | N/A |  | N/A |  | N/A |  |  |  |  |  |  |  |  |  |
| Calvi et al. (2022) |  | N/A |  | N/A |  | N/A |  |  |  |  |  |  |  |  |  |

Item fulfilled

Some concerns or unclear

Item not fulfilled

**Table S3.6 Level of evidence for adductor-related return-to-play (RTP) criteria**

| RTP criteria | Domain | Study | GRADE level of evidence | | | | | | |
| --- | --- | --- | --- | --- | --- | --- | --- | --- | --- |
|  |  |  | Starting level of evidence | Risk of bias | Inconsistency | Indirectness | Imprecise evidence | Publication bias | Level of evidence |
| No pain | Absence of clinical symptoms | Mechó et al. (2023) | Low |  |  |  |  |  | Very low |
|  | No pain at palpation | Serner et al. (2020a) | Low |  |  |  |  |  | Very low |
|  |  | Serner et al. (2021) |  |  |  |  |  |  |  |
|  |  | Serner et al. (2020b) |  |  |  |  |  |  |  |
|  | No pain during resisted contraction | Hölmich et al. (1999) | High |  |  |  |  |  | Moderate |
|  |  | Serner et al. (2020a) |  |  |  |  |  |  |  |
|  |  | Serner et al. (2021) |  |  |  |  |  |  |  |
|  |  | Serner et al. (2020b) |  |  |  |  |  |  |  |
|  | No pain during passive stretching | Serner et al. (2020a) | Low |  |  |  |  |  | Very low |
|  |  | Serner et al. (2021) |  |  |  |  |  |  |  |
|  |  | Serner et al. (2020b) |  |  |  |  |  |  |  |
|  | No pain during on-field/agility tasks | Serner et al. (2020a) | High |  |  |  |  |  | Moderate |
|  |  | Serner et al. (2021) |  |  |  |  |  |  |  |
|  |  | Serner et al. (2020b) |  |  |  |  |  |  |  |
|  |  | Nagamoto (2020) |  |  |  |  |  |  |  |
|  |  | Tak et al. (2020) |  |  |  |  |  |  |  |
|  |  | Jiménez-Rubio et al. (2021) |  |  |  |  |  |  |  |
|  |  | Hölmich et al. (1999) |  |  |  |  |  |  |  |
|  |  | Hägglund et al. (2007) |  |  |  |  |  |  |  |
| Football-specific abilities | Similar change of direction performance | Serner et al. (2020a) | Low |  |  |  |  |  | Very low |
|  |  | Serner et al. (2021) |  |  |  |  |  |  |  |
|  |  | Serner et al. (2020b) |  |  |  |  |  |  |  |
|  | Similar performance on technical–tactical tasks | Serner et al. (2020a) | Low |  |  |  |  |  | Very low |
|  |  | Serner et al. (2021) |  |  |  |  |  |  |  |
|  |  | Serner et al. (2020b) |  |  |  |  |  |  |  |
| External load progression | Completion of one full team training session | Serner et al. (2020a) | High |  |  |  |  |  | Moderate |
|  |  | Serner et al. (2021) |  |  |  |  |  |  |  |
|  |  | Serner et al. (2020b) |  |  |  |  |  |  |  |
|  |  | Nagamoto (2020) |  |  |  |  |  |  |  |
|  |  | Hägglund et al. (2007) |  |  |  |  |  |  |  |
|  | <10% difference of GPS-based performance in training session | Jiménez-Rubio et al. (2021) | Low |  |  |  |  |  | Very low |
| Strength | Similar hip adduction strength | Corazza et al. (2013) | Low |  |  |  |  |  | Very low |
|  | Similar isokinetic hamstring and quadriceps strength | Mechó et al. (2023) | Low |  |  |  |  |  | Very low |
|  |  | Corazza et al. (2013) |  |  |  |  |  |  |  |
| Muscle imaging | Maturity of the scar on MRI or ultrasound | Corazza et al. (2013) | Low |  |  |  |  |  | Very low |
|  |  | Jiménez-Rubio et al. (2013) |  |  |  |  |  |  |  |
|  | Absence of swelling | Hägglund et al. (2007) | High |  |  |  |  |  | Low |
| Healing | Adequate time of recovery according to the degree of the injury | Nagamoto (2020) | Low |  |  |  |  |  | Very low |
|  |  | Pezzota et al. (2018) |  |  |  |  |  |  |  |

**Table S3.7 Level of evidence for calf-related return-to-play criteria**

| RTP criteria | Domain | Study | GRADE level of evidence | | | | | | |
| --- | --- | --- | --- | --- | --- | --- | --- | --- | --- |
|  |  |  | Starting level of evidence | Risk of bias | Inconsistency | Indirectness | Imprecise evidence | Publication bias | Level of evidence |
| No pain | Absence of clinical symptoms | Pedret et al. (2015) | Low |  |  |  |  |  | Very low |
|  | No pain during on-field/agility tasks | Hägglund et al. (2007) | High |  |  |  |  |  | Low |
|  |  | Nagamoto (2020) |  |  |  |  |  |  |  |
|  | No pain during ambulation | Kwak et al. (2006) | Low |  |  |  |  |  | Very low |
| External load progression | Completion of one full team training session | Hägglund et al. (2007) | High |  |  |  |  |  | Low |
|  |  | Nagamoto (2020) |  |  |  |  |  |  |  |
| Muscle imaging | Absence of swelling | Hägglund et al. (2007) | High |  |  |  |  |  | Low |
| Healing | Adequate time of recovery according to degree of the injury | Nagamoto (2020) | Low |  |  |  |  |  | Very low |
|  |  | Pedret et al. (2015) |  |  |  |  |  |  |  |

**Table S3.8 Level of evidence for quadriceps-related return-to-play (RTP) criteria**

| RTP criteria | Domain | Study | GRADE level of evidence | | | | | | |
| --- | --- | --- | --- | --- | --- | --- | --- | --- | --- |
|  |  |  | Starting level of evidence | Risk of bias | Inconsistency | Indirectness | Imprecise evidence | Publication bias | Level of evidence |
| No pain | Absence of clinical symptoms | Balius et al. (2023) | High |  |  |  |  |  | Low |
|  |  | Shimozaki et al. (2022) |  |  |  |  |  |  |  |
|  |  | Bayer et al. (2018) |  |  |  |  |  |  |  |
|  | No pain during on-field/agility tasks | Valera-Garrido et al. (2020) | High |  |  |  |  |  | Low |
|  |  | Nagamoto (2020) |  |  |  |  |  |  |  |
|  |  | Bayer et al. (2018) |  |  |  |  |  |  |  |
|  |  | Hägglund et al. (2007) |  |  |  |  |  |  |  |
|  | No pain during and after repeated sprint ability | Bayer et al. (2018) | High |  |  |  |  |  | Low |
|  | No pain in single-leg jump | Bayer et al. (2018) | High |  |  |  |  |  | Low |
|  | No pain kicking | Valera-Garrido et al. (2020) | Low |  |  |  |  |  | Very low |
| Football-specific abilities | Similar performance on technical–tactical tasks | Shimozaki et al. (2022) | Low |  |  |  |  |  | Very low |
|  |  | Valera-Garrido et al. (2020) |  |  |  |  |  |  |  |
| External load progression | Completion of one full team training session | Nagamoto (2020) | High |  |  |  |  |  | Low |
|  |  | Hägglund et al. (2007) |  |  |  |  |  |  |  |
|  | <10% difference of GPS-based performance in training session | Valera-Garrido et al. (2020) | Low |  |  |  |  |  | Very low |
| Strength | Similar knee extension strength | Corazza et al. (2013) | Low |  |  |  |  |  | Very low |
|  | Similar isokinetic hamstring and quadriceps strength | Corazza et al. (2013) | Low |  |  |  |  |  | Very low |
| Muscle imaging | Maturity of the scar on MRI or ultrasound | Corazza et al. (2013) | Low |  |  |  |  |  | Very low |
|  | Absence of swelling | Hägglund et al. (2007) | High |  |  |  |  |  | Low |
| Healing | Adequate time of recovery according to degree of the injury | Nagamoto (2020) | High |  |  |  |  |  | Very low |
|  |  | Bayer et al. (2018) |  |  |  |  |  |  |  |

**Table S3.9 Level of evidence for unspecified lower limb muscle injury return-to-play (RTP) criteria**

| RTP criteria | Domain | Study | GRADE level of evidence | | | | | | |
| --- | --- | --- | --- | --- | --- | --- | --- | --- | --- |
|  |  |  | Starting level of evidence | Risk of bias | Inconsistency | Indirectness | Imprecise evidence | Publication bias | Level of evidence |
| No pain | Absence of clinical symptoms | Raya-González et al. (2019) | Low |  |  |  |  |  | Very low |
|  | No pain during on-field/agility tasks | Gómez-Piqueras et al. (2018) | Low |  |  |  |  |  | Very low |
|  |  | Bezuglov et al. (2022) |  |  |  |  |  |  |  |
| Football-specific abilities | Similar change of direction performance | Gómez-Piqueras et al. (2018) | Low |  |  |  |  |  | Very low |
|  | Similar performance on technical–tactical tasks | Gómez-Piqueras et al. (2018) | Low |  |  |  |  |  | Very low |
|  |  | Portillo et al. (2020) |  |  |  |  |  |  |  |
|  |  | Fuller et al. (2006) |  |  |  |  |  |  |  |
| External load progression | Completion of four full team training sessions | Bengtsson et al. (2019) | Low |  |  |  |  |  | Very low |
|  |  | Bezuglov et al. (2022) |  |  |  |  |  |  |  |
|  | Similar GPS-based performance in training session | Portillo et al. (2020) | Low |  |  |  |  |  | Very low |
|  | Similar sprinting demands | Portillo et al. (2020) | Low |  |  |  |  |  | Very low |
| Jumping performance | Similar countermovement jump performance | Gómez-Piqueras et al. (2018) | Low |  |  |  |  |  | Very low |
|  | Similar unilateral jump | Gómez-Piqueras et al. (2018) | Low |  |  |  |  |  | Very low |
|  | Similar triple hop | Gómez-Piqueras et al. (2018) | Low |  |  |  |  |  | Very low |
| Muscle imaging | Maturity of the scar on MRI or ultrasound | Calvi et al. (2022) | Low |  |  |  |  |  | Very low |
|  | Absence of swelling | Gómez-Piqueras et al. (2018) | Low |  |  |  |  |  | Very low |
| Psychology | Subjective readiness | Gómez-Piqueras et al. (2018) | Low |  |  |  |  |  | Very low |
|  | Low score in POMS questionnaire | Gómez-Piqueras et al. (2018) | Low |  |  |  |  |  | Very low |
| Anthropometry | <0.5% change in fat | Gómez-Piqueras et al. (2018) | Low |  |  |  |  |  | Very low |
| Movement quality | <2 cm asymmetry in Y balance test | Gómez-Piqueras et al. (2018) | Low |  |  |  |  |  | Very low |

Fulfillment: Level of evidence not downgraded for this outcome

Unclear fulfillment: Level of evidence not downgraded for this outcome

No fulfillment: Level of evidence downgraded by one level for this outcome

No fulfillment: Level of evidence downgraded by two levels for this outcome

Level of evidence was rated as high, moderate, low or very low [1]. Level of evidence started at high when RTP criteria were based on randomized controlled trials (RCTs i.e., 2 or more studies) or low if best evidence was based on longitudinal cohort studies [1]. Since RTP criteria is an outcome within the broader rehabilitation process, and no effect estimates exist for key outcomes (i.e., time to return to play and re-injuries), the level of evidence was assessed based on current recommendations for outcomes without effect sizes and using a narrative synthesis of the results [2]. An example of GRADE application for each domain based on existing evidence [2] can be found on Table S4.5. Level of evidence was downgraded if :1) most studies (i.e., >50%) that reported RTP criteria presented high risk of bias (downgraded by one level) or all RCTs presented high risk of bias (downgraded by two levels) according to the Grade handbook[1]; 2) there was inconsistency in specific RTP criteria across different domains (see Supplementary File 2); or 3) imprecision was found. Evidence was downgraded by one level if there were <800 participants in a comparison [3] and by two levels if there also was no clear direction of effects. All studies were pre-judged at low risk of indirectness, since the eligibility criteria used guaranteed relevant populations and interventions, and there was no need to use surrogate outcomes. Risk of publication bias could not be assessed due to the reduced number of studies (i.e., <10) available for each domain. When evidence was based on RCTs, the Risk of Bias 2 tool [4] was used; when evidence was based on longitudinal cohort studies, the Scottish Intercollegiate Guidelines Network checklist for cohort studies [5] was used. When evidence was based on cross-sectional, expert consensus, or case studies, the level of evidence was rated as very low.

**Table S3.10 An example of the application of modified GRADE for rating certainty of evidence of the studies reporting each RTP criterion based on previous recommendations [2].**

| Outcome: Absence of clinical symptoms following adductor injury | | |
| --- | --- | --- |
| GRADE domain | Judgement | Concerns about certainty of domains |
| Methodological limitations of the studies | Of the five studies reporting this criterion, only one is a longitudinal cohort study, showing a high risk of bias in detection bias. The remaining studies reporting this criterion are expert consensus studies or case studies. Notably, there is a high risk of bias in blinded evaluators. Therefore, following the recommendations of the SIGN Checklist for cohort studies, the overall risk of bias has been classified as high. | Serious |
| Inconsistency | The absence of clinical symptoms was measured in a very similar way in all the studies reporting this criterion. In addition, the scales used for assessment are similar (i.e., 0-10 scale) and the original sources referenced for the evaluation of this outcome are similar. | Not serious |
| Indirectness | Given our eligibility criteria and the created domains for assessment based on existing evidence, indirectness should be rated with no concerns. | Not serious |
| Imprecision | Studies reporting this criterion included less than 800 soccer players. | Serious |
| Publication bias | Publication bias could not be adequately assessed, as only one study citing this criterion reported key outcomes such as re-injuries or time to return to play, leaving no basis for comparison. | Not suspected |

**References:**

1. Schünemann H, Brożek J, Guyatt G, Oxman A. Handbook for grading the quality of evidence and the strength of recommendations using the GRADE approach. Updated October. 2013;2013:15.

2. Murad MH, Mustafa RA, Schünemann HJ, Sultan S, Santesso N. Rating the certainty in evidence in the absence of a single estimate of effect. Evidence Based Medicine. 2017;22:85–7.

3. Guyatt G, Oxman AD, Kunz R, Brozek J, Alonso-Coello P, Rind D, et al. Corrigendum to GRADE guidelines 6. Rating the quality of evidence-imprecision. J Clin Epidemiol 2011;64:1283–1293. J Clin Epidemiol. 2021;137:265.

4. Sterne JAC, Savović J, Page MJ, Elbers RG, Blencowe NS, Boutron I, et al. RoB 2: a revised tool for assessing risk of bias in randomised trials. BMJ. 2019;l4898.

5. Scottish Intercollegiate Guidelines Network (SIGN) Checklist. [cited 2023 Dec 10]; Available from: Scottish intercollegiate guidelines network (SIGN)

6. Silder A, Sherry MA, Sanfilippo J, Tuite MJ, Hetzel SJ, Heiderscheit BC. Clinical and Morphological Changes Following 2 Rehabilitation Programs for Acute Hamstring Strain Injuries: A Randomized Clinical Trial. Journal of Orthopaedic & Sports Physical Therapy. 2013;43:284–99.
